# Clinical Utility of Copy Number Abnormality Analysis in the Evaluation of Melanocytic Lesions for Diagnosis and Prognosis: An Evidence-Based Review from the Cancer Genomics Consortium Working Group for Melanocytic Lesions

**DOI:** 10.3390/genes17030331

**Published:** 2026-03-18

**Authors:** Cynthia Reyes Barron, Katherine B. Geiersbach, Ahmed K. Alomari, Kristen L. Deak, Shivani Golem, Eli S. Williams, Umut Aypar, Ying S. Zou, Lei Wei, Alka Chaubey, Nikhil Sahajpal, Ravindra Kolhe, Tanzy M. Love, Larry Prokop, M. Anwar Iqbal

**Affiliations:** 1 Pathology Professional Services, El Paso, TX 79902, USA; 2 Mayo Clinic, Rochester, MN 55905, USA; geiersbach.katherine@mayo.edu (K.B.G.); prokop.larry@mayo.edu (L.P.); 3 Department of Pathology and Laboratory Medicine, Indiana University School of Medicine, Indianapolis, IN 46202, USA; akalomar@iu.edu; 4 Department of Pathology, Health System Clinical Labs, Duke University, Durham, NC 27710, USA; kristen.deak@duke.edu; 5 Department of Pathology and Laboratory Medicine, University of Kansas Medical Center, Kansas City, KS 66160, USA; sgolem@kumc.edu; 6 Department of Pathology, University of Virginia School of Medicine, Charlottesville, VA 22903, USA; eliswilliams@proton.me; 7 Memorial Sloan Kettering Cancer Center, New York, NY 10065, USA; ayparu@mskcc.org; 8 Department of Pathology and Genetic Medicine, Johns Hopkins University School of Medicine, Baltimore, MD 21205, USA; 9 Department of Biostatistics and Bioinformatics, Roswell Park Comprehensive Cancer Center, Buffalo, NY 14263, USA; lei.wei@roswellpark.org; 10 Bionano Genomics, San Diego, CA 92121, USA; 11 Greenwood Genetic Center, Greenville, SC 29605, USA; nsahajpal@ggc.org; 12 Department of Pathology, Medical College of Georgia, Augusta University, Augusta, GA 30912, USA; rkolhe@augusta.edu; 13 Department of Biostatistics and Computational Biology, University of Rochester Medical Center, Rochester, NY 14642, USA; tanzy_love@urmc.rochester.edu; 14 Department of Pathology and Laboratory Medicine, University of Rochester Medical Center, Rochester, NY 14642, USA

**Keywords:** melanoma, ambiguous melanocytic lesions, comparative genomic hybridization, chromosomal microarray, fluorescence in situ hybridization, melanoma diagnosis, melanoma prognosis, copy number abnormality

## Abstract

**Background/Objective**: Although most melanocytic lesions are diagnosed as benign or malignant by histopathologic evaluation, with or without the aid of immunohistochemistry, diagnosis may remain uncertain in a minority of cases. Assessment of copy number abnormalities (CNAs) may provide sufficient additional evidence to favor either a benign or malignant diagnosis in both pediatric and adult cases and in melanocytic lesions of various subtypes, including Spitzoid, mucosal, and acral. CNAs are common in melanomas, while they are rare, with few exceptions, in benign lesions. Detection of CNAs by fluorescence in situ hybridization (FISH) and chromosomal microarray (CMA) has been well established for melanocytic lesions, with advantages and disadvantages for each. The objective of this meta-analysis was to evaluate the utility of CNA testing for the diagnosis of melanoma, across subtypes, when a lesion remains ambiguous after histopathologic and immunohistochemical assessment. In addition, the utility of CNAs to determine prognosis in established diagnoses of melanoma was also evaluated. **Methods**: The Cancer Genomics Consortium Working Group for Melanocytic Lesions reviewed published data from January 1998 through September 2022 of CNAs in melanocytic lesions detected by either FISH or CMA and conducted a meta-analysis of the findings. **Results**: Specific abnormalities common in primary cutaneous melanomas of various subtypes and uveal melanomas were enumerated. Differences in CNAs found in primary versus metastatic lesions were also determined, and published evidence for prognosis was summarized. **Conclusions**: The working group established evidence-based recommendations for the use of CNA testing for evaluation of ambiguous melanocytic lesions.

## 1. Introduction

### 1.1. Overview of Diagnostic Challenge

Benign melanocytic proliferations are extremely common, with most people possessing several benign melanocytic nevi or moles. Nevi may be present at birth or be acquired through adulthood and often regress later in life. Almost all will remain benign and never become malignant [1,2]. Melanoma is a complex malignancy arising from melanocytes. The estimate of new cases in the United States in 2025 is over 100,000 (https://seer.cancer.gov/statfacts/html/melan.html (accessed on 24 August 2025)). Sun exposure has long been recognized as a risk, yet damage from ultraviolet radiation is not always a factor in malignancy. The World Health Organization categorizes melanoma into nine pathways, only three of which are typically associated with cumulative solar damage. Precursor lesions and characteristic genetic mutations are considered along each pathway [3]. These pathways help our understanding of tumor biology and, in some cases, progression from benign precursor lesions to malignancy. Copy number abnormalities (CNAs), diffuse aneuploidy, and loss of heterozygosity may be important events in melanoma progression, with greater involvement in late-stage and metastatic disease [4].

**Figure 1:**
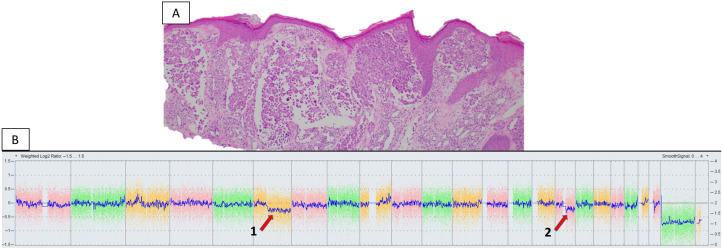
(**A**) Melanocytic lesion in 10-year-old male with atypical histopathologic features but not definitive for melanoma (photo courtesy of Glynis Scott, MD). (**B**) Chromosomal microarray with abnormal results demonstrating genomic alterations including loss of 6q11.1q27 (red arrow 1) and 16q11.2q24.3 (red arrow 2) (courtesy of Katherine Geiersbach, MD).

Both clinical history and dermoscopy can play a role in the initial evaluation of pigmented lesions, and the findings should be considered through further assessment [5]. Histopathologic evaluation of the biopsy tissue then serves to categorize a particular lesion as benign or malignant and either confirm or refute a clinical suspicion. In the vast majority of cases, histopathologic assessment, with or without the aid of immunohistochemical stains, is sufficient for diagnosis. However, a minority of cases possess features that are ambiguous and may be suspicious for malignant melanoma but not definitive (see Figure 1). This melanocytic lesion from a 10-year-old male displayed cytologic atypia, lack of maturation, and increased proliferative activity with scattered atypical mitoses on histopathologic evaluation. CMA was abnormal, demonstrating loss of 6q11.1q27 and loss of 16q11.2q24.3. The final diagnosis was malignant melanoma, Spitzoid type. Subsequently, the patient underwent sentinel lymph node biopsy, which demonstrated metastatic disease.

Targeted fluorescence in situ hybridization (FISH) and chromosomal microarray analysis (CMA)/comparative genomic hybridization (CGH) may add evidence for either a benign or malignant diagnosis [6]. The National Comprehensive Cancer Network (NCCN) guidelines list immunohistochemistry (IHC), comparative genomic hybridization (CGH), fluorescence in situ hybridization (FISH), gene expression profiling (GEP), single-nucleotide polymorphism array (aSNP), and next-generation sequencing (NGS) as ancillary tests that may help in the diagnosis of ambiguous melanocytic lesions. * The American Society of Dermatopathology (ASDP) appropriate use criteria for ancillary testing in melanocytic lesions are rated CGH/CMA, FISH, qRT-PCR, and *TERT* promoter mutation tests for 80 clinical scenarios. CGH/CMA and FISH were deemed to be usually appropriate in most scenarios involving melanocytic lesions that were suggestive or suspicious for melanoma but not definitive histopathologically [7]. However, classification of melanocytic lesions is not a simple dichotomy even with the aid of all recommended ancillary studies. There are lesions categorized as melanocytomas, with specific histopathologic and molecular findings and malignant potential that is intermediate between benign nevi and melanoma. Melanocytic tumors of uncertain malignant potential (MELTUMPs) are designated as such when the malignant potential is unknown and the histopathologic findings and molecular alterations are insufficient for a definitive diagnosis of melanoma [8]. An accurate classification, whether benign, malignant, intermediate, or uncertain, will guide clinical management appropriately.

* Referenced with permission from the NCCN Clinical Practice Guidelines in Oncology (NCCN Guidelines^®^) for Melanoma: Cutaneous V.3.2024. © National Comprehensive Cancer Network, Inc., Plymouth Meeting, PA, USA, 2024. All rights reserved. Accessed [25 November 2024]. To view the most recent and complete version of the guideline, go online to NCCN.org. NCCN makes no warranties of any kind whatsoever regarding their content, use or application and disclaims any responsibility for their application or use in any way.

### 1.2. Overview of FISH and CMA Techniques

FISH to detect chromosomal abnormalities, including CNAs, is a practical and widely used technique across malignancies. FISH analysis can detect and enumerate specific DNA sequences of targeted genes within cell or tissue samples by using fluorescent probes. This method allows for visualization of the interrogated loci but is typically limited to five or fewer probes per hybridization. FISH requires approximately 50–200 nuclei per probe set, either individual cells fixed on a glass slide or unstained FFPE tissue cut at approximately 3–4 microns, and has a limit of detection between 5 and 20% depending on sample type, abnormality, and probe design.

CMA is a high-resolution technique used to detect genetic imbalances, such as deletions, duplications, and chromosome aneuploidy, across the entire genome. The ability to utilize CMA using DNA extracted from formalin-fixed, paraffin-embedded tissue (FFPE) expanded the exploration of CNAs in melanomas over 25 years ago [9]. Unlike cytogenetic testing for other malignancies, the necessity of CNA evaluation for melanocytic lesions is usually determined after tissue has been processed, fixed in formalin, embedded in paraffin, and assessed histopathologically. Microarray DNA requirements vary by platform but typically require 10 consecutive unstained, ~5 micron-thick sections of FFPE tissue placed on positively charged slides and require approximately 30% lesional cells. The interpretation of CNAs may vary slightly by laboratory. In general, ≥1 megabases (Mb) for deletions, ≥2 Mb for copy gains, and ≥10 Mb for loss of heterozygosity are considered adequate for reporting abnormalities, although changes in fewer Mb are acceptable if involving clinically relevant genes [10].

### 1.3. Clinical Application of FISH Versus CMA

The four-probe FISH set, including 6p25, 6q23, CEP6, and 11q13, was established by Gerami et al. (2009) [11] and has been shown to detect melanoma with high sensitivity and specificity [12]. Gerami determined that detection of CNAs using that limited set of probes provided excellent distinction between benign and malignant lesions [13]. Its utility has been tested extensively in cutaneous melanomas of various subtypes. The set was subsequently expanded to improve sensitivity with the inclusion of probes for 8q24 and 9p21. The methodology and criteria for defining abnormalities have varied slightly depending on the laboratory, with similar conclusions—FISH distinguishes benign nevi from melanoma with high specificity and sensitivity [14]. Although results in ambiguous lesions may still be challenging and tetraploidy may give false positive results [15], there are many advantages to using FISH to identify CNAs in melanocytic lesions.

FISH can be performed on limited samples without the need to purify DNA [16], which may be challenging in some cases. Because FISH is performed on a tissue section and abnormalities can be assessed in different regions of the same lesion, it has the potential to discriminate between benign and malignant components of a melanocytic proliferation. FISH abnormalities have been detected in regions of melanocytic proliferations that are malignant, while the benign components in the same lesions have no abnormalities, affording the means to stage melanomas more accurately [17,18]. This ability to discriminate between populations has also provided insight into melanoma tumorigenesis. Dysplastic nevi appear to possess some abnormalities, while melanomas arising in dysplastic nevi acquire additional CNAs [18].

However, FISH requires skill to interpret, and the heterogeneity in melanomas may further complicate interpretation, making FISH difficult to automate [19]. Also, melanomas presenting with specific histopathologic characteristics are challenging to evaluate using FISH. For example, CMA seems to have improved sensitivity in detecting superficial spreading melanoma than FISH [20]. FISH may also be more challenging in lesions where the melanocytes are growing in a sclerotic stroma [21]. Furthermore, detection of CNAs using CMA may be more sensitive and specific than FISH in lesions with borderline features, using a cutoff of >3 or ≥3 CNAs for favoring malignant [22,23]. The criteria for counting CNVs are detailed in a review by Ebbelaar et al. [23]. Briefly, aberrations including gains, losses, amplifications, chromothripsis, copy-neutral loss of heterozygosity and chromosomal fusions each count as one, while homozygous losses count as two. Although aneuploidy is counted as one by Ebbelaar, the recommendation proposed by this group excludes counting whole-chromosome gains and losses for cutaneous melanomas given that they are relatively common in benign melanocytic neoplasms, particularly in pediatric patients. These alterations are well documented in proliferative nodules arising within congenital melanocytic nevi [24,25]. Importantly, such large-scale chromosomal copy number changes do not appear to confer adverse biologic behavior in these settings. Accordingly, they are often discounted when enumerating copy number alterations in cutaneous melanocytic tumors. In contrast, copy number changes in atypical and malignant melanocytic cutaneous neoplasms more often reflect underlying genomic instability or crisis, resulting in numerous segmental gains and losses rather than isolated whole-chromosome events. From a biologic standpoint, one would, therefore, not expect isolated whole-chromosome gains or losses to carry the same diagnostic or prognostic significance in this context.

In one study, the sensitivity and specificity for definitive melanomas were 82.4% and 100%, respectively, using FISH and 97.1% and 100%, respectively, using CMA [10]. In the same study, the sensitivity dropped in ambiguous lesions to 47.1% using FISH and 61.2% using CMA [10]. Because CMA has the advantage of assessing the entire genome, while FISH is limited by specific probes, sensitivity and specificity may be improved. Lesions that are negative by FISH may show aberrations by CMA [26]. Most melanomas are associated with sun exposure, and melanomas arising in sun-protected sites may harbor different CNAs [27]; thus, a limited probe set may miss abnormalities at such sites. It is important to note that CMA is also subject to false-negative results. As CMA analyzes the entire cell population of the submitted tissue, CNAs in specimens with a low tumor percentage may not meet the detection threshold, even with microdissection. DNA extraction and purification may be challenging in cases with abundant melanin pigment [28]. Additionally, the size resolution of a particular CMA may exclude potentially significant intragenic CNAs from detection. These assay limitations must be carefully considered when interpreting a negative CMA result [29].

The decision to use either FISH or CMA to assess an ambiguous melanocytic lesion largely depends on availability of the test, adequacy of the specimen, and preference of the pathologist and clinician. CMA, with the capability of analyzing CNAs across the entire genome, is now often preferred. CMA analysis has shown utility in both pediatric and adult populations for analyzing ambiguous lesions, including melanoma arising in large congenital nevi [30,31]. More recently, digital PCR has been documented to yield sensitivity comparable to FISH for targeted CNA detection and may provide an inexpensive alternative if CMA testing is not available [32,33,34].

Given the potential importance of CNA analysis in melanocytic lesions, the Cancer Genomics Consortium Melanoma Working Group set out to review published research with an aim to evaluate the diagnostic utility and clinical significance of CNA analysis. This evidence-based review encompasses the findings. For the purposes of this review, CNAs detected by FISH and CMA were considered in aggregate. While the working group acknowledges differences in sensitivities and resolution of FISH and CMA approaches, as described in the preceding sections, aggregation of CNA data provides a more comprehensive review of the existing literature. Both techniques have demonstrated clinical utility for CNA detection in melanocytic lesions. The genetic landscape of melanoma is complex. Mutations and translocations were not assessed in this review, although certain mutations, such as *TERT* promoter mutations, may also be helpful in distinguishing benign from malignant lesions, and some mutations may be associated with specific CNAs [35].

## 2. Methods

Extensive literature searches were conducted for peer-reviewed manuscripts in the English language published from January 1998 through September 2022 through Ovid. Criteria included all manuscripts with findings of copy number abnormalities in melanocytic lesions. Publications with research conducted only on cell lines or animal models were excluded. Case reports and manuscripts with germline CNAs associated with melanoma predisposition were also excluded. In addition, manuscripts with CNAs detected by methods other than CMA or FISH and manuscripts reporting results only from cases in The Cancer Genome Atlas (TCGA) were excluded. A total of 516 manuscripts were assessed, and 235 were retrieved and deemed eligible for inclusion after thorough review by working group members (see Supplementary Figure S1 and Supplementary Table S13) [36]. Working group members assessing individual manuscripts evaluated study quality and risk of bias, and recommendations for exclusion/inclusion were evaluated by other working group members during the selection process. Manuscripts eligible for inclusion were assigned to individual working group members for review and data collection. Data was further reviewed by the primary author (CRB) and any issues were discussed in regular meetings of working group members. The findings of individual manuscripts were tabulated, the number of published cases with a given CNA was summarized, and statistical analysis was performed resulting in a comprehensive meta-analysis. (The meta-analysis is not registered).

The findings were tabulated in aggregate and by specific melanoma subtype, including general cutaneous, uveal, Spitzoid, nevoid, desmoplastic, blue nevus like, mucosal, and acral melanomas, as well as findings in benign melanocytic nevi, when available. Amplifications and gains were combined, as were deletions and losses, to accommodate for differences in reporting between the publications. Only cases with a final diagnosis of benign or malignant were included, regardless of how that final diagnosis was reached. In some cases, the diagnosis was obtained by histopathologic evaluation with or without the aid of immunohistochemistry, and in others, CNA analysis played a role. Lesions with a diagnosis that remained ambiguous or uncertain were not tabulated. Atypical Spitzoid tumors/Spitz melanocytomas were an exception as the diagnosis is considered a definitive category.

We aimed to present the findings from numerous studies in aggregate. One of the challenges was drawing conclusions when the results from the various studies were reported differently. Some manuscripts reported regions on chromosome arms only (i.e., gain of 11p), while others reported findings implicating a specific gene (i.e., 11p15-*HRAS*). When the locus of an abnormality was reported differently, separate entries were tabulated for statistical purposes. Also, the CMA platforms and FISH probes used to identify aberrations varied. For statistical analysis, identical CNAs were tabulated together regardless of the platform used to identify them.

For each proportion of interest, a 95% confidence interval was calculated by inverting the score test. For the finding of many by CMA, a one-sided lower bound for the population proportion was calculated by the same method. When a proportion was tested to see if it was common (at least 5%), a one-sample test for proportions with Yates continuity correction was performed against the null hypothesis of 5% prevalence. When two proportions were compared, for example, gains compared to losses, a two-sample test for proportions with continuity correction was performed against the null hypothesis that the two proportions were equal. For all statistical tests, ɑ = 0.05 was used as the threshold of significance.

## 3. Results

### 3.1. Overview

**Figure 2:**
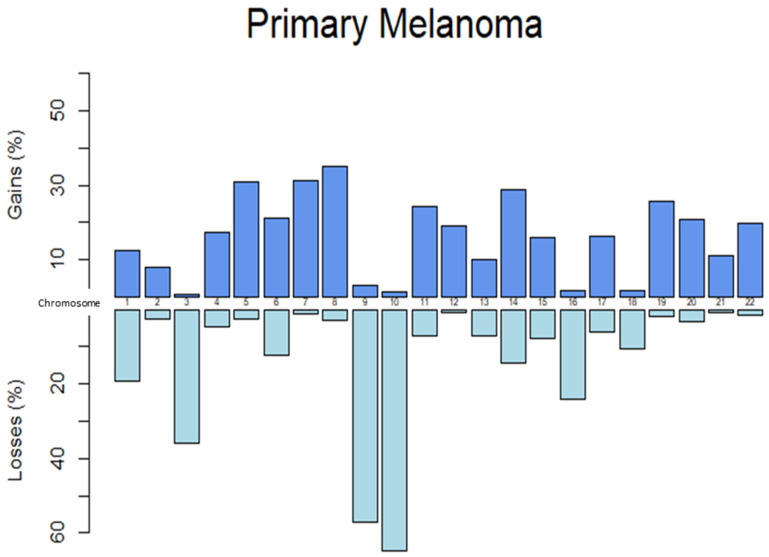
Rates of gains and losses across each chromosome in primary cutaneous melanoma of all subtypes combined.

Overall, gains were significantly more prevalent than losses in general cutaneous, desmoplastic, mucosal, and acral melanoma, while losses were more prevalent in uveal melanoma (all *p* < 0.005, see Supplementary Table S1). The rates (percentage of cases) reporting gains and losses were also tabulated across each chromosome for primary melanoma of all subtypes combined (see Supplementary Table S2 and Figure 2). The chromosome with the highest rate of gain was chromosome 8, likely due to commonly seen gain in 8q24. The chromosome with the highest rate of loss was chromosome 10, likely due to commonly seen monosomy and separate losses in both 10p and 10q. Gains and losses in specific chromosome arms and regions reported in significantly greater than 5% of cases and published in three or more manuscripts were tabulated (see Table 1 and Table 2). The region with the greatest rate of gain was 6p25 (including *RREB1*) in 58% (95% CI 55–61%) of melanomas. Although 6q25 (*ARID1B*) had the highest rate of loss overall at 44% (95% CI 34–55%), it was only reported in mucosal melanomas. Loss of 9p21 (*CDKN2A*) had the second highest rate of loss at 38.1% (95% CI 36–40%) and was reported in general cutaneous, uveal, Spitzoid, nevoid, mucosal, and acral melanomas at high rates.

### 3.2. Ocular Melanoma

Uveal melanoma is rare, comprising only 3–5% of all melanomas, with an incidence of approximately 5 cases per million [37,38]. It includes malignancies in the iris, choroid, and ciliary body. CNAs in uveal melanoma have been well studied and carry important prognostic significance. NCCN guidelines recommend molecular or chromosomal testing when a biopsy is performed at the time of diagnosis or on resected tumors to determine prognostic information. The guidelines specify that disomy 3 and gain of chromosome 6p are seen in cases with low risk of metastases, while cases with monosomy 3 and gain of 8q are high risk. * The guidelines are based on a large body of research with patient outcomes. This review confirmed these findings. The results of chromosome 3 ploidy, detected by FISH or CMA, have been published for thousands of cases. Monosomy 3 was found in 49% (95% CI 48–50%) of uveal melanomas [39,40,41,42,43,44,45,46,47,48,49,50,51,52,53,54,55,56,57,58,59,60,61,62,63,64,65,66,67,68,69,70,71,72,73,74,75,76,77,78,79,80,81,82,83,84,85,86,87,88,89,90,91,92,93,94,95,96,97,98,99,100,101,102,103,104,105,106,107,108,109], while whole-chromosome gains (polysomy 3) were reported very rarely [43,60,81,96]. Partial loss of chromosome 3 was seen in approximately 8% (95% CI 7–9%) of cases [51,79,85,101,110,111,112,113]. Monosomy 3 was consistently associated with a worse prognosis and greater risk of metastasis. Monosomy 3 was related to extraocular extension [63] and larger tumor size [73].

* Referenced with permission from the NCCN Clinical Practice Guidelines in Oncology (NCCN Guidelines^®^) for Melanoma Uveal V.1.2024. © National Comprehensive Cancer Network, Inc. 2024. All rights reserved. Accessed [25 November 2024]. To view the most recent and complete version of the guideline, go online to NCCN.org. NCCN makes no warranties of any kind whatsoever regarding their content, use or application and disclaims any responsibility for their application or use in any way.

Tumor heterogeneity in uveal melanoma is common [62,95]. Although the higher percentage of cells with loss of chromosome 3 appears to indicate worse patient outcomes [114], detection of monosomy 3 in greater than 5% of tumor cells may be sufficient for increased risk of death due to metastasis [71]. In cases with partial loss, the loss of *BAP1* at 3p21 was associated with a worse prognosis than cases without this loss [75,93]. *BAP1* may play a critical role in uveal melanoma tumorigenesis because suppression of expression or mutation of this gene was associated with monosomy 3 [75,76]. On the other hand, 6p gain, an abnormality that occurs in 37% (95% CI 35–39%) of uveal melanomas, was rarely seen in tumors with monosomy 3 and was associated with a low risk of metastasis and less aggressive clinical behavior [39,41,42,44,45,51,52,60,61,64,67,68,74,89,96,98,101,105,107,115,116,117,118,119,120]. In particular, amplifications or gains of 6p21 including *CDKN1A* or 6p25 including *RIPK1* or *FOXC1* may play a role in improved outcomes [53,94,113]. Gain of 6q was seen in only 5% (95% CI 4–7%) of cases but was also associated with improved outcomes [43,51,60,61,64,74,96,101,105].

Many studies have reported abnormalities of chromosome 8 in uveal melanoma. Polysomy 8, gains of 8q, isochromosome 8q, and both losses and gains of 8p were independently associated with a high risk of metastasis and worse outcomes (see Supplementary Table S3). Specifically, gains of *MYC* have been reported in 58% (95% CI 52–64%) of uveal melanomas and were associated with worse outcomes. Loss of 1p and gain of 1q were also associated with a worse prognosis (see Supplementary Tables S3 and S4). Gain of 1q is infrequent overall but may be a frequent occurrence in liver metastases [45]. Studies have shown that monosomy 3 observed in combination with other abnormalities, including loss of 1p and gain of chromosome 8, results in a worse prognosis than monosomy 3 alone.

CNAs common in uveal melanomas are distinct from those in cutaneous and mucosal melanomas. This distinction can be exploited when determining primary tumor site in metastases. Detecting monosomy 3 is more indicative of a uveal primary because it is rare in general cutaneous melanoma [55]. Monosomy 3 was reported in only one study in cutaneous melanoma at a rate of 6% (95% CI 0–29%) [10]. Monosomy 3 has also been detected in circulating tumor cells in patients with uveal melanoma and may be a non-invasive method of assessing disease and risk of metastasis [56]. Although gain of 8q was more common in uveal melanoma, 52% (95% CI 51–54%), than general cutaneous melanoma, 26% (95% CI 20–33%), the frequency is high for both.

Conjunctival melanomas harbor CNAs similar to cutaneous and mucosal melanomas and should be considered distinct from uveal melanomas [121].

### 3.3. General Cutaneous Melanoma

Most cutaneous melanomas have detectable CNAs, and with few exceptions, benign melanocytic nevi do not. In 1998, Bastian et al. determined that 94% of melanomas have CNAs and suspected that technical difficulties had prohibited detection from the remaining 6% [9]. Many subsequent studies have confirmed the significant difference in CNAs between benign nevi and cutaneous melanomas. In our analysis, FISH testing yielded abnormal results in 82% (95% CI 80–84%) of cutaneous melanomas when different probe sets were considered in aggregate (see Supplementary Table S10). The number of cases with published results was close to 1700 (see Table 3). Of the standard targeted regions, 6p25, 6q23, 8q24, 9p21, and 11q13, the most common CNA reported was gain at 6p25, present in 58% (95% CI 54–62%) of general cutaneous melanomas, followed by loss of 9p21, present in 52% (95% CI 49–55%) of published cases (see Table 4). In the general cutaneous melanoma category, 94% (95% CI 92–96%) of melanomas were deemed to have abnormal results favoring malignancy by CMA (see Table 5). A result was deemed likely indicative of malignancy if greater than three CNAs were detected or if there were fewer than three but at least one CNA had been determined to be common in melanomas.

Reportedly, benign nevi that demonstrate no atypia have chromosomal abnormalities in less than 1% of cases, while those with atypical features may harbor CNAs [122]. Gains, losses, abnormal FISH results, and abnormal CNA results by CMA were not significantly greater than 5% for benign cutaneous nevi in this study (see Supplementary Table S9). A gain of 6p25 was reported in three manuscripts in a total of four nevi out of 115 nevi studied for a rate of 3% (95% CI 1–9%) [10,123,124,125]. Whether these nevi demonstrated histopathologic atypia is unknown. The long-term clinical outcomes are also unknown. Some special classes of melanocytic neoplasms have shown more than three CNVs but still had an overall indolent behavior and did not reach morphologic criteria for a malignant diagnosis. Examples include tumors with a *MAP2K1* in-frame deletion [126].

### 3.4. Spitzoid Lesions

Spitzoid lesions range from benign spindled and epithelioid cell nevi or Spitz nevi to atypical Spitzoid tumors/Spitz melanocytomas to malignant Spitzoid melanomas/Spitz melanomas. They can be some of the most difficult to classify on histopathologic grounds alone. Moreover, the terms Spitz melanoma and Spitzoid melanoma have been used interchangeably in the literature; however, they represent two molecularly distinct groups of neoplasms according to the latest WHO classification [127]. Benign lesions can display cellular atypia and other concerning features such as mitoses and Pagetoid spread. Ancillary studies can be very helpful for definitive diagnosis [128]. Previously described as melanoma of childhood, molecular studies have helped elucidate differences between benign Spitz nevi and melanoma [129]. These lesions, including Spitzoid melanomas, are more common in young patients than older adults. Chromosomal analysis of pediatric lesions has demonstrated that malignant lesions in this population also carry CNAs common to melanomas in adults, particularly homozygous loss of *CDKN2A* [130,131]. The original four-probe FISH set (6p25, 6q23, CEP6, 11q13) was suboptimal when applied to Spitzoid lesions. Addition of probes at 9p21 and CEP9 improved the sensitivity from 70% to 85% in one study [132]. A probe set with 6p25, 11q13, 9p21 and 8q24 demonstrated an overall 94% sensitivity and 98% specificity in another study [133], with improvement in detecting malignant Spitz lesions. When the results of testing with common FISH panels were considered in aggregate, the sensitivity for Spitzoid/Spitz melanoma was significantly less than for general cutaneous melanoma, with a rate of 70% (95% CI 62–77%) in our analysis (see Table 3).

As in general cutaneous melanomas, the most common CNA was gain of 6p25 (54%, 95% CI 43–64%), followed by loss of 9p21 (39%, 95% CI 31–48%) (see Table 4 and Supplementary Table S5). Gains in 6p25 or 11q13 or homozygous loss of 9p21 may have a significant association with aggressive behavior [134].

**Figure 3:**
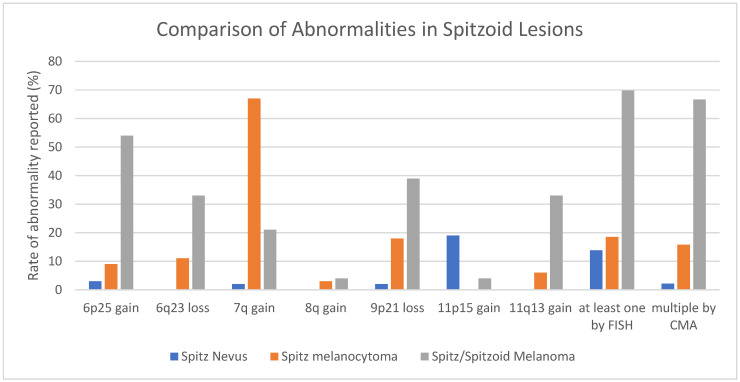
Comparison of rates of abnormalities reported in Spitzoid lesions. See also Supplementary Table S5.

The rate of abnormalities detected by FISH in benign Spitz nevi was much greater than in other cutaneous nevi. At least one abnormality was detected by FISH in 14% (95% CI 10–19%) of benign Spitz nevi and 19% (95% CI 15–22%) of Spitz melanocytomas. Likewise, CNAs by CMA were higher in Spitz lesions than in other subtypes, with 2% (95% CI 0.1–13%) of benign Spitz nevi and 16% (95% CI 4–40%) of Spitz melanocytomas demonstrating multiple CNAs. Although CNAs are generally rare in benign Spitz nevi, one exception is isolated gain of 11p, which includes *HRAS*. In one study of 54 benign nevi, the only abnormality observed was gain of 11p in seven nevi, all of which had Spitzoid morphology [135]. We tabulated gain of 11p in 19% (95% CI 15–24%) of benign Spitz nevi and 4% (95% CI 0.7–16%) of Spitzoid/Spitz melanomas (see Supplementary Table S5 and Figure 3). This finding suggests that the Spitzoid/Spitz melanomas with this abnormality may have arisen from benign Spitz nevi.

In addition to gain of 11p including *HRAS*, copy state transitions (gains or losses) over kinase genes, including *ALK*, *BRAF*, *ERBB4*, *FGFR1*, *MAP3K3*, *MAP3K8*, *MERTK*, *MET*, *NTRK1*, *NTRK2*, *NTRK3*, *PRKDC*, *RET*, and *ROS1*, can be identified on CMA studies of Spitz neoplasms, and the finding of a CNA disrupting a kinase gene can point to an oncogenic fusion in one of these genes [3,128,136,137,138,139]. However, because CMA does not detect balanced rearrangements, it is not the ideal method for detecting gene fusions.

Studies of Spitzoid lesions in pediatric cases with clinical follow-up call into question the definition of benign and malignant. When clinical follow-up is available, pediatric patients seem to do well, even when their Spitzoid lesions carry chromosomal aberrations and when sentinel lymph nodes are positive [140]. When the diagnosis of Spitzoid/Spitz melanoma is rendered in a pediatric patient, treatment and follow-up may need to be tailored with the difference to adults in mind.

*BAP1*-inactivated melanocytomas were formerly considered part of the Spitzoid family of lesions [141]. The discussion of these melanocytomas is beyond the scope of this review. Like other melanocytomas, their malignant potential is considered low or intermediate between benign and malignant [8]. Yet, *BAP1* loss may be seen in melanomas, including blue nevus-like melanoma [142], and loss of *BAP1* in a melanocytic lesion should prompt a thorough clinical history and consideration of germline testing for *BAP1* tumor predisposition syndrome.

Pigmented spindle cell nevi (of Reed) are another subtype of melanocytic lesion that are usually considered part of the Spitzoid family. CNVs in these lesions are not well studied, but pigmented spindle cell nevi may also be distinguished from malignant melanoma with pigmented and spindled cells by the paucity of CNAs in the former, as demonstrated in one study utilizing FISH (original four-probe set—6p25, 6q23, CEP6, 11q13) that found CNAs in only 1 of 15 pigmented spindle cell nevi [123].

### 3.5. Nevoid Melanoma

Nevoid melanomas are uncommon, comprising less than 1% of all melanomas, and can be diagnostically challenging because they look deceptively benign both clinically and histopathologically [143]. Less data has been published about this melanoma subtype, and less is known about this subtype’s genomic aberrations than others. However, FISH panels were abnormal in 93% (95% CI 84–98%) of published cases (see Table 3). Loss of 9p21 was the most common CNA. Multiple CNAs (>3) by CMA were detected in 85% (95% CI 54–97%) of these melanomas (see Table 5).

Proliferative nodules arising in congenital nevi are other entities with a difficult diagnostic classification and are often assessed together with nevoid melanomas. Like melanoma, they may have increased mitotic activity and increased CNAs; however, they are rare, and limited data is available. Several reports have been published regarding the utility of CNA analysis in distinguishing benign from malignant; taken together, the available data suggests that distinguishing whole-chromosome versus intrachromosomal copy number abnormalities by CMA may aid in the accurate diagnosis of proliferative nodules and distinguish them from nevoid melanomas [24,25,144].

### 3.6. Desmoplastic Melanoma

Desmoplastic melanoma is associated with high ultraviolet radiation exposure and consists of spindle cells coursing through a fibrotic, scar-like stroma [3]. The malignant melanocytes may be subtle histopathologically and confused with fibroblasts in scar tissue. Detection of abnormalities by FISH may be more challenging in lesions such as these, with their distinct morphology [145,146]. By CMA, 86% (95% CI 56–97%) of cases demonstrated multiple CNAs (see Table 5).

### 3.7. Blue Nevi and Related Lesions

Like desmoplastic melanoma, the melanocytes in the blue nevus category of lesions usually consist of cells with a spindle morphology. However, these lesions are usually pigmented and have abundant melanophages. Blue nevus-like melanomas are rare. CMA has demonstrated utility in distinguishing between benign and malignant in this category of melanocytic lesions [147]. In this analysis, 80% (95% CI 61–92%) of blue nevus-like melanomas had greater than three CNAs by CMA (see Table 5). FISH was abnormal in 94% (95% CI 68–100%) of blue nevus-like melanomas (original four-probe set—6p25, 6q23, CEP6, 11q13) (see Table 3).

### 3.8. Mucosal Melanoma

Studies have shown that melanoma arising at mucosal sites, whether genital, oral, or sinonasal, are molecularly distinct from most cutaneous melanomas, perhaps because tumorigenesis is not related to chronic sun damage at these sites [148,149,150,151,152]. Gain of 4q12 harboring *KIT* was seen more frequently in mucosal melanomas (31%, 95% CI 26–37%) than melanomas at cutaneous sites (6%, 95% CI 4–8%) and may have implications for treatment. Although mutations in *KIT* make tumors susceptible to targeted therapy, gains may make them resistant [153]. The rate of gain of 6p25 was higher for mucosal melanomas than any other subtype in this study, with a rate of 97% (95% CI 82–100%, see Table 4). FISH was abnormal in 100% of 30 cases reported (using a six-probe FISH set, including 6p25, 6q23, CEP6, 9p21, CEP9, and 11q13), and CMA detected greater than three abnormalities in 95% (95% CI 72–100%) of cases (see Table 3 and Table 5).

### 3.9. Acral Melanoma

**Figure 4:**
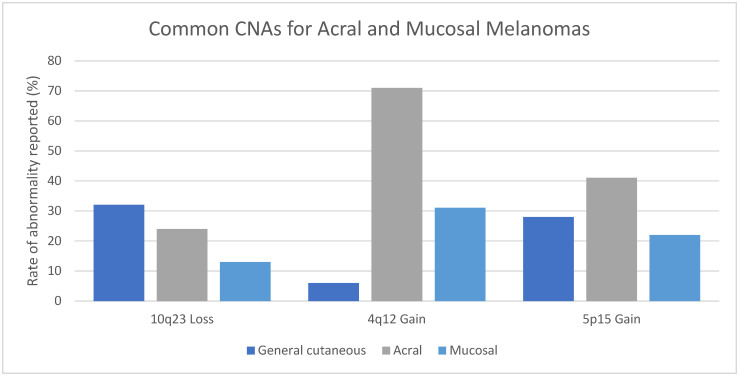
Differences in rates of CNAs in 10q23 (*PTEN*), 4q12 (*KIT*), and 5p15 (*TERT*) in acral and mucosal melanomas compared to general cutaneous melanomas.

Awareness of the differences in CNAs between mucosal, acral and cutaneous melanomas from other sites may be helpful when evaluating challenging lesions [154]. Melanomas occurring at acral sites demonstrate CNAs that involve chromosomes 5p, 11q, and 12q more often than melanomas at other sites [135]. Gains of 5p15 encompassing *TERT* were reported in 41% (95% CI 38–45%) of acral melanomas and only 28% (95% CI 22–36%) of general cutaneous melanomas (see Figure 4). Moreover, gains were more common in acral melanomas than in melanomas at other sites [154]. This observation was confirmed by our study, which showed significantly more gains (33%, 95% CI 32–35%) than losses (7%, 95% CI 6.5–8.4%) in acral melanomas (*p* < 0.001). Differences between mucosal, acral, and melanoma at other cutaneous sites may also be seen in 10q23 and 5p15 (see Figure 4).

The four-probe FISH set has been tested specifically in acral melanomas, and the results demonstrate high sensitivity for these lesions [14]. In this study, FISH was abnormal in 88% (95% CI 81–92%) of acral melanomas (see Table 3), and abnormalities were detected by CMA in 100% of published cases (see Table 5).

### 3.10. Implications for Prognosis and Treatment

As knowledge of the impact of CNAs in melanoma grows, questions arise regarding not only the utility for diagnosis but also the implications they may carry for prognosis and treatment. Recent studies with clinical follow-up paired with CNA analysis in both primary and metastatic lesions in the same patient are providing important evidence for the role of CNAs. However, to our knowledge, there have been no large-scale prospective studies to evaluate the impact of CNAs in melanocytic lesions.

As expected, both primary melanomas and metastatic melanomas harbor CNAs that can be detected by FISH and CMA. The rates between primary and metastatic were similar, and there were no significant differences (see Supplementary Table S6). Yet, the detection of CNAs by FISH may be independently, significantly associated with aggressive behavior and worse outcomes [155]. The number of CNAs may also be correlated with clinical behavior, with a greater number associated with more aggressive behavior in some studies [156,157,158]. The presence of significant aneuploidy and loss of heterozygosity have been detected in advanced disease [4].

One form of CNA observed in a subset of malignancies, including melanomas, is chromothripsis or a pattern of clustered CNAs on a single chromosome arm or subregion or sometimes multiple chromosome arms or subregions. Chromothripsis was originally described in 2011 [159] and has been associated with a poor prognosis in one study of 20 melanomas, where chromothripsis or complex copy alterations were associated with reduced overall survival [160]. A more recent study of cancers using whole-genome sequencing, which included 106 melanomas, showed that over half of melanomas had evidence of chromothripsis, suggesting that this type of alteration is oncogenic in at least a subset of melanomas [161]. Chromothripsis is a mechanism for activation of kinase genes in Spitz melanocytic neoplasms via complex structural rearrangements that generate oncogenic fusions involving kinase genes, many of which are targetable with clinically approved kinase inhibitor therapies [162,163,164,165]. There is currently insufficient evidence to support enumerating genomic segments of chromothriptic gains or losses on CMA in order to determine the likelihood that a melanocytic neoplasm is malignant; however, accumulating evidence shows that chromothripsis promotes tumor progression via activation of oncogenes and inactivation of tumor suppressor genes [166,167,168].

Multiple studies have focused on a specific abnormality in an attempt to elucidate which may be more common in metastases and, therefore, imply a worse prognosis when detected in a primary lesion. For example, one study concluded that deletion of 1p36 was more common in metastatic lesions [169]. Our analysis demonstrated a similar rate: 34% (95% CI 27–42%) in primary cutaneous melanomas and 35% (95% CI 27–45%) in metastases. Gains of *PREX1* on 20q13 were shown to indicate lower metastasis-free survival in one study [170]. Our data showed similar rates of 20q13 gains in primary (27%, 95% CI 21–33%) and metastatic lesions (26%, 95% CI 17–37%). In contrast, gains at 7q34 harboring *BRAF* were more common in metastatic lesions (58%, 95% CI 49–66%) than primary melanomas (30%, 95% CI 26–35%). Copy number gains may be associated with *BRAF* mutations [171], and gains may be associated with a shorter time to disease progression [172].

Gains at 8q24 (*MYC*) and 11q13 (*CCND1*), both common, were reportedly associated with more aggressive behavior and metastases [173,174,175,176]. Gains of 1q (reported in 45% of metastases and 29% of primary cutaneous melanomas) and 6p (reported in 50% of metastases and 40% of primary cutaneous melanomas) may also predict lower survival [177]. Losses of 6q (reported in 50% of metastases and 27% of primary cutaneous melanomas) and 10q (reported in 40% of metastases and 39% of primary cutaneous melanomas) were associated with a poor prognosis and/or ulceration [178]. *EGFR* amplification/polysomy 7 may also be associated with greater tumor thickness and more aggressive behavior [179,180,181]. Polysomy 7 was seen in 57% of metastases and 25% of primary cutaneous lesions in our study (see Supplementary Table S7). When gains of *EGFR* were reported specifically, they demonstrated a rate of 34% in metastases and 17% in primary cutaneous lesions, supporting an association with more aggressive behavior (see Supplementary Table S7). Multiple studies have shown that increased *PHIP* copy number on 6q14 is associated with metastasis and decreased survival [182,183,184]. However, many additional gains and losses may be implicated [185,186], and our analysis found no reports of *PHIP* gains in metastases, although the rate of gain in primary lesions was 34%.

**Figure 5:**
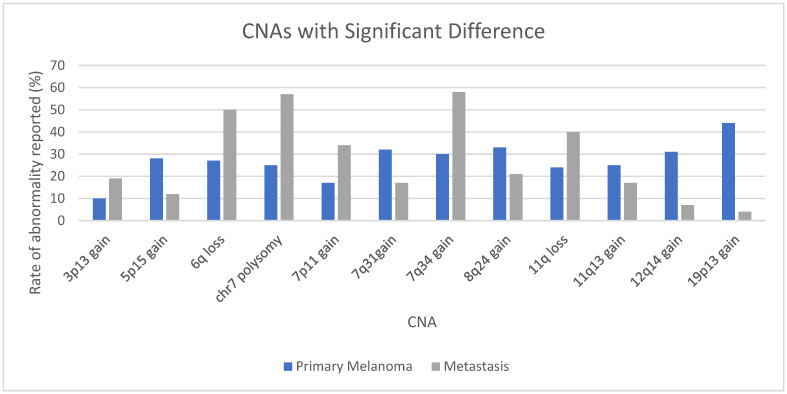
CNAs with significant difference in rates between primary melanomas and metastases reported by 3 manuscripts or more in at least 50 cases each in general cutaneous melanomas. See also Supplementary Table S7.

**Figure 6:**
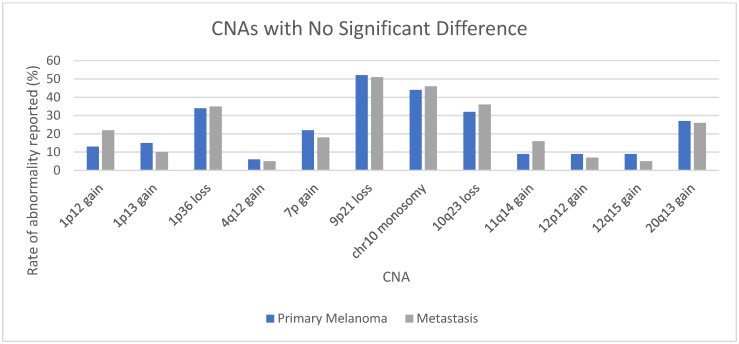
CNAs with no significant difference in rates between primary melanomas and metastases reported by 3 manuscripts or more in at least 50 cases each in general cutaneous melanomas. See also Supplementary Table S8.

To better understand the differences between metastases and primary melanomas, a comparison was made of CNAs in general cutaneous melanomas with the inclusion criteria of being reported by at least three manuscripts in at least 50 cases each of primary melanoma and metastases. There was a significant difference in the rates in 12 CNAs. Half of these were more common in metastases, including gains of 3p13 (*MITF*), 7p11 (*EGFR*), and 7q34 (*BRAF*) (see Supplementary Table S7 and Figure 5). The same inclusion criteria identified 12 CNAs with no significant difference between primary and metastatic lesions, including loss of 1p36, loss of 9p21 (*CDKN2A*), and loss of 10q23 (*PTEN*) (see Supplementary Table S8 and Figure 6).

Treatment of aggressive and metastatic melanoma is continuously under investigation. Mutations are routinely assessed for targeted therapy. The question remains whether CNAs should be evaluated routinely as well. *TERT* copy number gain, more common in acral melanomas (41% vs. 28% in general cutaneous melanomas), may be indicative of response to therapy [187]. Although immune checkpoint inhibitors have made a significant impact in treating advanced stage melanomas, some patients progress. A study found that *MDM2*, *MDM4*, and *EGFR* amplifications were not associated with progression in patients with mucosal or acral melanomas on immune checkpoint inhibitors [188]. However, copy number gains of 12q14 including *CDK4* were more common in patients resistant to anti-PD1 therapy [189]. CNAs of *PDL1* on 9p24 were uncommon; losses were seen in 10% of cases across subtypes (see Table 2), and studies have shown they do not predict response to anti-PD1 targeted therapy [190]. Targeted therapy in melanomas harboring *MET* amplifications is under investigation [191]; we found a rate of 26% for this CNA in all subtypes. As targeted therapies emerge across malignancies, those associated with CNAs should be considered when applicable to melanomas.

### 3.11. Other Ancillary Techniques

Immunohistochemistry has been a valuable tool used by pathologists to assess melanocytic lesions for many years. Specifically, stains that are positive in melanocytes, such as SOX10, S100, and Melan-A/MART1, can help pathologists evaluate architectural features and identify malignant characteristics, such as asymmetry, Pagetoid spread, and dermal invasion. Stains for p16, Ki-67 and HMB45 are often evaluated in conjunction with determination of staining patterns indicative of malignancy, and studies have shown a high concordance with cytogenetic results [192,193,194]. PRAME is a newer stain that may also have diagnostic utility. For challenging lesions, the specificity of PRAME staining may be as high as 95%; however, the sensitivity may be only 75%, and positive staining in benign Spitz nevi is a potential hazard with interpretation [195,196]. Studies have shown a concordance of 90% between PRAME staining and cytogenetic studies in ambiguous melanocytic lesions [195]. The accessibility, low cost, rapid turnaround time and ease of IHC make it primary among ancillary tests. When the results remain ambiguous, evaluation of CNAs should be considered. For challenging cases, cytogenetics provides additional information that, when used in conjunction with histopathologic and IHC results, may yield improved diagnoses [193].

The ASDP also indicates that testing for TERT promoter mutations may have utility in determining malignancy in ambiguous melanocytic lesions. The specificity may be as high as 98.6%, while the sensitivity is lower at 77.9%, and the frequency of this mutation may vary by melanoma subtype [197,198].

Gene expression profiling (GEP) may also serve as a useful ancillary tool for diagnosis. In one study, it showed 80% concordance with FISH in unambiguous cases and 73% concordance in ambiguous cases; however, FISH results showed better concordance with histopathologic assessment (97% vs. 83%) [199]. In another study, FISH and GEP showed 69% agreement in ambiguous lesions, and CMA demonstrated better correlation with morphologic interpretation than either FISH or GEP [200]. Yet, agreement with FISH was 50.9% and 57.1% with CMA when ambiguous lesions were included [201]. Additional research is necessary to determine the validity of GEP across melanoma subtypes. For example, in desmoplastic melanoma, GEP and CMA were discordant in four cases out of nine [202].

Detection of CNAs in melanocytic lesions by CMA and FISH is well established. Publications using methods other than CMA and FISH were excluded from this review. However, there is increasing evidence that NGS and MLPA can accurately detect CNAs, and these platforms may have certain advantages for clinical use [203]. For example, NGS testing is able to assess copy number changes in addition to SNVs; therefore, it has the potential to replace SNP-array testing of melanoma in the future.

## 4. Discussion

**Figure 7:**
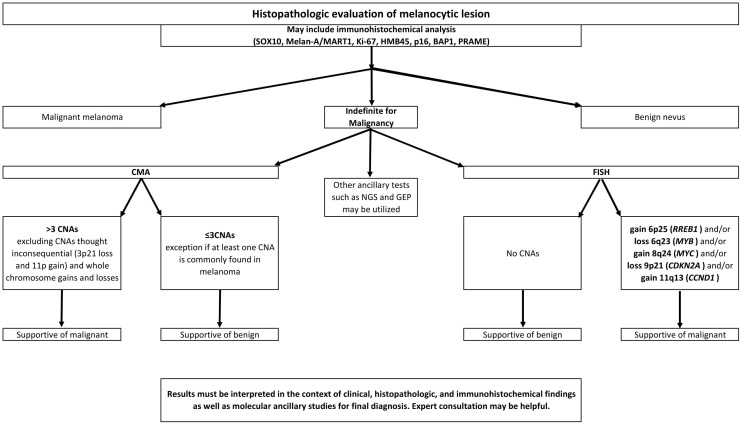
Algorithmic approach to the evaluation of melanocytic lesions and ancillary testing for CNAs.

Inaccuracies in melanoma diagnosis can carry critical implications for patient management. Ample studies have shown that cytogenetic analyses in melanocytic lesions are valuable for malignancy determination and, in many cases, prognosis determination. In aggregate, the total number of gains, losses, and FISH abnormalities was uncommon in benign nevi. They were not significantly greater than 5% (see Supplementary Table S9). While CNAs were present in the majority of malignant melanomas detected by either FISH or CMA across subtypes, there are differences in CNA findings between melanoma subtypes, which may be a reflection of variations in the molecular pathways involved in tumorigenesis and the involvement of damage caused by ultraviolet radiation. An algorithmic approach to the use of CNA in the evaluation of melanocytic lesions is suggested in Figure 7, applicable across subtypes.

In assessing conclusions from hundreds of publications in the field, several limitations were encountered. Cases that remained ambiguous after all diagnostic tools were applied were not counted in the aggregate data. Yet, CNA analysis in ambiguous lesions is the most pertinent for daily practice and patient care, as these are the lesions that are most difficult to diagnose on histopathologic grounds alone. Whether or not a final diagnosis was rendered before or after the incorporation of CNA results, cases were included in statistical analysis together, which may have resulted in incorporation bias.

Because the dichotomous view of melanocytic lesions as benign or malignant may be too biologically simple, some studies favored classifying lesions on a spectrum [204]. It is important to remember that all findings must be considered in conjunction. The number of CNAs alone does not render a diagnosis of melanoma. A recent study of seven melanocytic lesions with Spitzoid morphology and amplification of a mutant NRAS gene concluded that all seven lesions were indefinite for melanoma, although additional CNAs were present in all cases [205]. When reported, the results for dysplastic nevi were considered benign. We found insufficient data on CNAs in dysplastic nevi to reach significance in classification into mild, moderate, and severe atypia. Several studies have shown that severely dysplastic nevi have more aberrations than mild and moderately dysplastic nevi but fewer than melanoma [16,122].

Conclusions from evidence-based review in this study are limited by the paucity of follow-up data. The treatment of melanoma with complete excision with negative margins is often curative for early-stage tumors. These may be the most difficult tumors to diagnose histopathologically because the early and superficial melanomas may not be clearly demonstrating the full spectrum of histopathologic abnormalities characteristic of malignancy. Positive outcomes, when present, may be attributed to early diagnosis and adequate treatment as well as prognostically less aggressive melanomas.

Although our literature search was extensive, all searches are limited by parameters and search engines. Furthermore, new research continues to emerge. Fortunately, with each new publication, awareness of CNA assessment to aid in diagnosis of melanocytic lesions is increasing among pathologists and dermatologists.

## 5. Evidence-Based Recommendations

- CMA or FISH do not need to be performed on all melanocytic lesions to assess malignancy. Those lesions considered ambiguous after assessment of histopathologic features, including immunohistochemical studies, may be good candidates for evaluation of CNAs (see Figure 7).
- For uveal melanomas, CNA studies for prognosis are validated and established.
- Although prognostic information may be gained by the evaluation of CNAs in primary cutaneous melanomas, routine testing has not been established.
- Differences in common CNAs between uveal and primary cutaneous melanomas may be exploited in determining the origin of metastases.
- CMA may be preferred to FISH because of its ability to assess the entire genome. FISH may be more helpful in small lesions where DNA extraction may yield insufficient material.
- FISH panels including probes for 6p25, 6q23, 8q24, 9p21, and 11q13 are reasonable and provide adequate sensitivity and specificity in most cases. Results are supportive of malignancy when at least one recurring CNA is detected.
- When results of FISH studies are inconclusive or negative, CMA, with the ability to identify abnormalities throughout the genome, may be performed.
- CMA is considered supportive of malignant melanoma when more than three CNAs are detected or when fewer CNAs are detected but those identified include an abnormality that is common in melanoma (>5%, see Table 1 and Table 2). CMA with three or fewer CNAs does not completely exclude a diagnosis of melanoma, and CMA with greater than three CNAs does not definitively diagnose melanoma.
- As with all testing modalities, limitations exist with CMA and FISH testing, and negative results do not completely exclude the presence of CNAs. Tumor heterogeneity, morphology, and percentage of tumor cells in a sample may limit detection.
- No single ancillary study is sufficient for diagnosis. Results must be considered in conjunction with clinical presentation and histopathologic findings. Evaluation of CNAs may add support to favor either a benign or malignant diagnosis. Although CNA testing adds evidence, a definitive diagnosis must include all other results in conjunction. Some lesions may remain ambiguous after all ancillary testing is performed and expert consultation is obtained.

## Figures and Tables

**Table 1:** Rates of gains significantly higher than 5% for specific chromosome regions in primary melanoma of all subtypes combined. All *p*-values in the listed CNAs were ≤0.05 and were considered indicative of significance. The abnormalities were reported by at least 3 manuscripts. Possible genes affected by the gain are listed.

Region	Rate of Gain (%)	Possible Genes Affected
1p12	12	*NOTCH2* ***, *ADAM30* ***
1p13	9	*NRAS* *
1q	26	
1q21	16	*PDE4DIP* ***, *BCL9* *, *S100A9* ***, *S100A10* ***, *S100A11* ***, *S100A12* ***
1q23	28	*NTRK1* *, *KIRREL* ***
1q32	41	*MDM4* *
2q31	14	*PDE11A* ***
3p13	10	*MITF* *
4p	10	
4q12	17	*KIT* *, *KDR* *, *PDGFRA* *
5p	14	
5p15	32	*TERT* *
5q	10	
6p	30	
6p25	58	*RREB1* *
6p21	25	*CCND3* *
6q14	34	*PHIP* ***
7p	23	
7p11	17	*EGFR* *
7q	27	
7q31	26	*MET* *
7q34	27	*BRAF* *
8p	6	
8q	50	
8q24	39	*MYC* *
11p15	15	*HRAS* *
11q	8	
11q13	27	*CCND1* *
11q14	11	*GAB2* *
12p12	8	*KRAS* *, *PIK3C2G* ***
12q14	21	*CDK4* *
12q15	11	*HDM2/MDM2* *
13q14	10	*RB1* **
14q32	37	*AKT1* *
15q	14	
17p13	18	*TP53* **
17q	18	
17q11	15	*NF1* **
17q24	32	*BPTF* ***, *PRKCA* *, *PRKAR1A* **
19p13	37	*MAP2K2* *
20p11	11	
20q	23	
20q13	22	*MYBL2* *, *ZNF217* *, *CYP24* ***, *STK6* *, *P-REX1* *, *SS18L1* ***, *GNAS* *, *SNAI1* *, *SNAI2* *
21q	14	
22q13	21	*MKL1* ***, *EP300* ***

* Oncogene; ** Tumor suppressor gene; *** Other/complex (context-dependent function, dual role, limited melanoma-specific evidence, or gene located within amplified locus without definitive driver status). Please see Supplementary Table S11.

**Table 2:** Rates of losses significantly higher than 5% for specific chromosome regions in primary melanoma of all subtypes combined. All *p*-values in the listed CNAs were ≤0.05 and were considered indicative of significance. The abnormalities were reported by at least 3 manuscripts. Possible genes affected by the loss are listed.

Region	Rate of Loss (%)	Possible Genes Affected
1p	23	
1p36	32	*PRDM16* ***, *ARID1A* **
3p21 ^a^	15	*BAP1* **
3q	14	
4q	10	
5q	22	
6q	14	
6q23	29	*MYB* ***
6q25 ^b^	44	*ARID1B* **
8p	9	
9p	32	
9p21	38	*CDKN2A* **
9p24	10	*CD274* ***, *JAK2* ***, *PTPRD* **
9q	29	
9q12	13	
10p	19	
10p15	14	*PRKCQ* ***, *NET1* ***, *KLF6* **, *IL15RA* ***, *CALML5* ***, *LARP4B* ***
10q	34	
10q23	25	*PTEN* **
11p11	23	*CD82* **
11q	18	
11q22	9	*YAP1* ***
11q24	26	*ETS1* ***, *CHEK1* ***
13q14	8	*RB1* **
16p	14	
16q	25	
16q23	13	*BANP* **, *CBFA2T3* **, *FANCA* **, *CDK10* ***
17p	21	
17p13	12	*TP53* **
18q	9	
20p11	18	
20q11	20	*E2F1* ***

^a^ May be inconsequential in melanocytic proliferations with Spitzoid morphology; ^b^ Reported only in mucosal melanomas; ** Tumor suppressor gene; *** Other/complex (context-dependent function, dual role, limited melanoma-specific evidence, or gene located within amplified locus without definitive driver status). Please see Supplementary Table S12.

**Table 3:** Rates of abnormalities detected by FISH panels for each melanoma subtype and 95% confidence intervals for the given rates. The number of cases on which the rates are based is given.

Melanoma Subtype	Rate of FISH Abnormality Detected (%)	Lower 95% Confidence Limit	Upper 95% Confidence Limit	Number of Cases
General cutaneous	82	80	84	1682
Acral	88	81	92	153
Blue nevus like	94	68	100	16
Mucosal	100	86	100	30
Nevoid	93	85	98	75
Spitzoid/Spitz	70	62	77	169

**Table 4:** Rates in percentage of CNAs commonly tested on FISH panels across different melanoma subtypes. Values in red represent rates calculated from <25 reported cases. The number of reported cases appears in parentheses (). The symbol “-” designates data not available.

Melanoma Subtype	Rate of Gain of 6p25	Rate of Loss of 6q23	Rate of Gain of 8q24	Rate of Loss of 9p21	Rate of Gain of 11q13
General cutaneous	58 (644)	38 (515)	33 (567)	52 (880)	25 (1871)
Acral	72 (149)	42 (171)	47 (79)	28 (222)	39 (515)
Blue nevus like	83 (23)	61 (23)	-	-	50 (18)
Desmoplastic	44 (16)	33 (3)	-	-	31 (16)
Mucosal	97 (33)	80 (20)	75 (24)	39 (233)	17 (260)
Nevoid	66 (41)	15 (41)	31 (13)	69 (13)	24 (41)
Spitzoid/Spitz	54 (99)	33 (141)	-	39 (134)	33 (146)
Uveal	-	33 (40)	58 (249)	-	0 (83)

**Table 5:** The percentage of melanomas with greater than 3 CNAs reported by CMA for each melanoma subtype and the 95% confidence lower bound for the proportion in the given number of reported cases.

Melanoma Subtype	Percentage of Cases with >3 CNAs by CMA (%)	Lower 95% Confidence Limit	Number of Cases
Overall	94		769
General cutaneous	94	92	579
Acral	100	96	83
Blue nevus like	80	64	30
Desmoplastic	86	61	14
Mucosal	95	76	19
Nevoid	85	58	13
Spitzoid/Spitz	67	16	3
Uveal	100	88	28

## Data Availability

The data presented in this study are available on request from the corresponding author due to the breadth of data acquired in the extensive review and meta-analysis.

## Supplementary Materials

The following supporting information can be downloaded at: https://www.mdpi.com/article/10.3390/genes17030331/s1, Supplementary Figure S1: PRISMA 2020 flow diagram for systematic reviews including searches of databases. Supplementary Table S1: Rates of gains and losses across melanoma subtypes combined. *p*-values are from a two-sample test of whether there is a difference between gains and losses. Supplementary Table S2: Rates of gains and losses for each chromosome in melanoma across all subtypes combined. *p*-values designate whether the rate of CNA is significantly higher than 5%. Significant values are in bold. Also shown are the number of manuscripts reporting results in CNAs for a given chromosome on which the rates are based. Also see Figure 2. Supplementary Table S3: Rates of chromosomal abnormalities detected in primary uveal melanoma associated with high risk of metastasis and aggressive clinical behavior. Supplementary Table S4: Rates of abnormalities for uveal primary and metastatic melanomas. *p*-values are for whether rates of the type of abnormality are significantly different between primary and metastatic uveal melanomas. Also shown are the number of manuscripts on which the rates are based. Values in red represent rates calculated from <25 reported cases. Supplementary Table S5: Comparison of rates of abnormalities reported in Spitzoid lesions in three or more manuscripts. See also Figure 3. The symbol “-” designates data not available. Supplementary Table S6: Rates of abnormalities for cutaneous primary and metastatic melanomas. *p*-values are for whether rates of the type of abnormality are significantly different between primary and metastatic melanomas. Also shown are the number of manuscripts on which the rates are based. Supplementary Table S7: CNAs reported in at least 3 manuscripts in at least 50 cases of primary melanomas and 50 cases of metastases with significant difference in rates (*p*-values given for differences). Individual *p*-values indicate whether each abnormality is significantly greater than 5%. Rates in bold were the greater of the comparison between primary and metastatic melanomas. See also Figure 5. Supplementary Table S8: CNAs reported in at least 3 manuscripts in at least 50 cases of primary melanomas and 50 cases of metastases with no significant difference in rates (*p*-values given for differences). Individual *p*-values indicate whether each abnormality is significantly greater than 5%. See also Figure 6. Supplementary Table S9: Abnormalities reported in various benign nevus subtypes. The 95% confidence intervals for each proportion of the abnormality are given. The symbol “-” designates data not available. Supplementary Table S10: FISH probe sets for analysis of melanocytic lesions with published data included in this study. Supplementary Table S11: Genes classified as other/complex in Table 1. Supplementary Table S12: Genes classified as other/complex in Table 2. Supplementary Table S13: Literature search strategy detail through Ovid. Database(s): EBM Reviews—Cochrane Central Register of Controlled Trials July 2022, EBM Reviews—Cochrane Database of Systematic Reviews 2005 to 31 August 2022, Embase 1974 to 2 September 2022, Ovid MEDLINE(R) and Epub Ahead of Print, In-Process, In-Data-Review & Other Non-Indexed Citations, Daily and Versions 1946 to 2 September 2022. Table S14. PRISMA 2020 Checklist.

## Abbreviations

The following abbreviations are used in this manuscript:

ASDP	American Society of Dermatopathology
aSNP	Single-Nucleotide Polymorphism Array
CGH	Comparative Genomic Hybridization
CI	Confidence Interval
CMA	Chromosomal Microarray
CNA	Copy Number Abnormality
DNA	Deoxyribonucleic Acid
FFPE	Formalin-Fixed, Paraffin-Embedded Tissue
FISH	Fluorescence In Situ Hybridization
GEP	Gene Expression Profiling
IHC	Immunohistochemistry
Mb	Megabases
MELTUMP	Melanocytic Tumors of Uncertain Malignant Potential
MLPA	Multiplex Ligation-Dependent Probe Amplification
NCCN	National Comprehensive Cancer Network
NGS	Next-Generation Sequencing
TCGA	The Cancer Genome Atlas

## References

[1] Elder D.E. (2006). Precursors to melanoma and their mimics: Nevi of special sites. Mod. Pathol..

[2] Tucker M.A. (2009). Melanoma epidemiology. Hematol. Oncol. Clin. N. Am..

[3] Elder D.E., Bastian B.C., Cree I.A., Massi D., Scolyer R.A. (2020). The 2018 World Health Organization Classification of Cutaneous, Mucosal, and Uveal Melanoma: Detailed Analysis of 9 Distinct Subtypes Defined by Their Evolutionary Pathway. Arch. Pathol. Lab. Med..

[4] Vergara I.A., Mintoff C.P., Sandhu S., McIntosh L., Young R.J., Wong S.Q., Colebatch A., Cameron D.L., Kwon J.L., Wolfe R. (2021). Evolution of late-stage metastatic melanoma is dominated by aneuploidy and whole genome doubling. Nat. Commun..

[5] Nardone B., Martini M., Busam K., Marghoob A., West D.P., Gerami P. (2012). Integrating clinical/dermatoscopic findings and fluorescence in situ hybridization in diagnosing melanocytic neoplasms with less than definitive histopathologic features. J. Am. Acad. Dermatol..

[6] Mesbah Ardakani N., Harvey N.T., Wood B.A. (2019). Polypoid Compound Melanocytic Proliferations: A Clinicopathological Study. Am. J. Dermatopathol..

[7] Fung M.A., Vidal C.I., Armbrecht E.A., Andea A.A., Cassarino D.S., Comfere N.I., Emanuel P.O., Ferringer T., Hristov A.C., AUC Committee Members (2022). Appropriate use criteria for ancillary diagnostic testing in dermatopathology: New recommendations for 11 tests and 220 clinical scenarios from the American Society of Dermatopathology Appropriate Use Criteria Committee. J. Cutan. Pathol..

[8] Duncan L.M., Elder D.E., Piepkorn M.W., Knezevich S.R., Blokx W.A.M., Bosenberg M., Busam K.J., Carr R., Cook M.G., Gerami P. (2025). Shifts in Cutaneous Melanocytic Tumor Diagnostic Terminology: Melanocytoma, MPATH-Dx V2.0 and the WHO Skin5. J. Cutan. Pathol..

[9] Bastian B.C., LeBoit P.E., Hamm H., Brocker E.B., Pinkel D. (1998). Chromosomal gains and losses in primary cutaneous melanomas detected by comparative genomic hybridization. Cancer Res..

[10] Geiersbach K.B., Gliem T.J., Jenkins S.M., Gaitatzes A.G., Brodersen P.R., Negro M.E., Clees M.J., Swanson K.E., Boeckman R.M., Natrop T.J. (2022). Single-Nucleotide Polymorphism Array for Histologically Ambiguous Melanocytic Tumors. J. Mol. Diagn..

[11] Gerami P., Jewell S.S., Morrison L.E., Blondin B., Schulz J., Ruffalo T., Matushek P.t., Legator M., Jacobson K., Dalton S.R. (2009). Fluorescence in situ hybridization (FISH) as an ancillary diagnostic tool in the diagnosis of melanoma. Am. J. Surg. Pathol..

[12] Gerami P., Geraminegad P. (2010). Fluorescence in situ hybridization as a diagnostic modality in melanocytic neoplasma. G. Ital. Dermatol. Venereol..

[13] Gerami P., Wass A., Mafee M., Fang Y., Pulitzer M.P., Busam K.J. (2009). Fluorescence in situ hybridization for distinguishing nevoid melanomas from mitotically active nevi. Am. J. Surg. Pathol..

[14] Lai Y., Wu Y., Liu R., Lu A., Zhou L., Jia L., Diao X., Li Z. (2020). Four-color fluorescence in-situ hybridization is useful to assist to distinguish early stage acral and cutaneous melanomas from dysplastic junctional or compound nevus. Diagn. Pathol..

[15] Zembowicz A., Yang S.E., Kafanas A., Lyle S.R. (2012). Correlation between histologic assessment and fluorescence in situ hybridization using MelanoSITE in evaluation of histologically ambiguous melanocytic lesions. Arch. Pathol. Lab. Med..

[16] Zimmermann A.K., Hirschmann A., Pfeiffer D., Paredes B.E., Diebold J. (2010). FISH analysis for diagnostic evaluation of challenging melanocytic lesions. Histol. Histopathol..

[17] Newman M.D., Lertsburapa T., Mirzabeigi M., Mafee M., Guitart J., Gerami P. (2009). Fluorescence in situ hybridization as a tool for microstaging in malignant melanoma. Mod. Pathol..

[18] Casorzo L., Luzzi C., Nardacchione A., Picciotto F., Pisacane A., Risio M. (2005). Fluorescence in situ hybridization (FISH) evaluation of chromosomes 6, 7, 9 and 10 throughout human melanocytic tumorigenesis. Melanoma Res..

[19] Uguen A., Uguen M., Talagas M., Gobin E., Marcorelles P., De Braekeleer M. (2016). Fluorescence in situ hybridization testing of chromosomes 6, 8, 9 and 11 in melanocytic tumors is difficult to automate and reveals tumor heterogeneity in melanomas. Oncol. Lett..

[20] Kutzner H., Metzler G., Argenyi Z., Requena L., Palmedo G., Mentzel T., Rutten A., Hantschke M., Paredes B.E., Scharer L. (2012). Histological and genetic evidence for a variant of superficial spreading melanoma composed predominantly of large nests. Mod. Pathol..

[21] Grcar-Kuzmanov B., Bostjancic E., Bandres J.A.C., Pizem J. (2018). Sclerosing Melanocytic Lesions (sclerosing Melanomas with Nevoid Features and Sclerosing Nevi with Pseudomelanomatous Features)—An Analysis of 90 Lesions. Radiol. Oncol..

[22] Carter M.D., Durham A.B., Miedema J.R., Harms P.W., Chan M.P., Patel R.M., Lowe L., Fullen D.R., Hristov A.C., Wang M. (2019). Molecular testing of borderline cutaneous melanocytic lesions: SNP array is more sensitive and specific than FISH. Hum. Pathol..

[23] Ebbelaar C.F., Jansen A.M.L., Bloem L.T., Blokx W.A.M. (2021). Genome-wide copy number variations as molecular diagnostic tool for cutaneous intermediate melanocytic lesions: A systematic review and individual patient data meta-analysis. Virchows Arch..

[24] Bastian B.C., Xiong J., Frieden I.J., Williams M.L., Chou P., Busam K., Pinkel D., LeBoit P.E. (2002). Genetic changes in neoplasms arising in congenital melanocytic nevi: Differences between nodular proliferations and melanomas. Am. J. Pathol..

[25] Yelamos O., Arva N.C., Obregon R., Yazdan P., Wagner A., Guitart J., Gerami P. (2015). A comparative study of proliferative nodules and lethal melanomas in congenital nevi from children. Am. J. Surg. Pathol..

[26] Kerl K., Palmedo G., Wiesner T., Mentzel T., Rutten A., Scharer L., Paredes B., Hantschke M., Kutzner H. (2012). A proposal for improving multicolor FISH sensitivity in the diagnosis of malignant melanoma using new combined criteria. Am. J. Dermatopathol..

[27] Chernoff K.A., Bordone L., Horst B., Simon K., Twadell W., Lee K., Cohen J.A., Wang S., Silvers D.N., Brunner G. (2009). GAB2 amplifications refine molecular classification of melanoma. Clin. Cancer Res..

[28] Price K., Linge C. (1999). The presence of melanin in genomic DNA isolated from pigmented cell lines interferes with successful polymerase chain reaction: A solution. Melanoma Res..

[29] Mikhail F.M., Biegel J.A., Cooley L.D., Dubuc A.M., Hirsch B., Horner V.L., Newman S., Shao L., Wolff D.J., Raca G. (2019). Technical laboratory standards for interpretation and reporting of acquired copy-number abnormalities and copy-neutral loss of heterozygosity in neoplastic disorders: A joint consensus recommendation from the American College of Medical Genetics and Genomics (ACMG) and the Cancer Genomics Consortium (CGC). Genet. Med..

[30] Verzi A.E., Bubley J.A., Haugh A.M., Zhang B., Wagner A., Kruse L., West D.P., Wayne J., Guitart J., Gerami P. (2017). A single-institution assessment of superficial spreading melanoma (SSM) in the pediatric population: Molecular and histopathologic features compared with adult SSM. J. Am. Acad. Dermatol..

[31] Lacoste C., Avril M.F., Frassati-Biaggi A., Dupin N., Chretien-Marquet B., Mahe E., Bodemer C., Vergier B., de la Fouchardiere A., Fraitag S. (2015). Malignant Melanoma Arising in Patients with a Large Congenital Melanocytic Naevus: Retrospective Study of 10 Cases with Cytogenetic Analysis. Acta Derm. Venereol..

[32] Ramos-Rodriguez A.J., McFadden J.R., Momtahen S., LeBlanc R.E., Yan S., Chaudhari A.S., Cloutier J.M., Stevanovic M., Barney R., Syku M. (2024). A novel method to assess copy number variations in melanocytic neoplasms: Droplet digital PCR for precise quantitation of MYC and MYB genes. J. Cutan. Pathol..

[33] McFadden J.R., Syku M., Barney R.E., Stevanovic M., Chaudhari A.S., O’Hern K.J., Chambers M., Baker C.M., LeBlanc R.E., Doan L. (2023). A Novel Method to Detect Copy Number Variation in Melanoma: Droplet Digital PCR for Quantitation of the CDKN2A Gene, a Proof-of-Concept Study. Am. J. Dermatopathol..

[34] O’Hern K., Barney R., Chambers M., Baker C., Stevanovic M., Tsongalis G.J., Hughes E., Sriharan A. (2023). A novel method to assess copy number variation in melanoma: Droplet digital PCR for precise quantitation of the RREB1 gene in formalin-fixed, paraffin-embedded melanocytic neoplasms, a proof-of-concept study. J. Cutan. Pathol..

[35] Lazar V., Ecsedi S., Vizkeleti L., Rakosy Z., Boross G., Szappanos B., Begany A., Emri G., Adany R., Balazs M. (2012). Marked genetic differences between BRAF and NRAS mutated primary melanomas as revealed by array comparative genomic hybridization. Melanoma Res..

[36] Page M.J., McKenzie J.E., Bossuyt P.M., Boutron I., Hoffmann T.C., Mulrow C.D., Shamseer L., Tetzlaff J.M., Akl E.A., Brennan S.E. (2021). The PRISMA 2020 statement: An updated guideline for reporting systematic reviews. BMJ.

[37] Xu Y., Lou L., Wang Y., Miao Q., Jin K., Chen M., Ye J. (2020). Epidemiological Study of Uveal Melanoma from US Surveillance, Epidemiology, and End Results Program (2010–2015). J. Ophthalmol..

[38] Kaliki S., Shields C.L. (2017). Uveal melanoma: Relatively rare but deadly cancer. Eye.

[39] de Lange M.J., van Pelt S.I., Versluis M., Jordanova E.S., Kroes W.G., Ruivenkamp C., van der Burg S.H., Luyten G.P., van Hall T., Jager M.J. (2015). Heterogeneity revealed by integrated genomic analysis uncovers a molecular switch in malignant uveal melanoma. Oncotarget.

[40] van Gils W., Mensink H.W., Kilic E., Vaarwater J., Verbiest M.M., Paridaens D., Luyten G.P., de Klein A., Bruggenwirth H.T. (2007). Expression of APITD1 is not related to copy number changes of chromosomal region 1p36 or the prognosis of uveal melanoma. Investig. Ophthalmol. Vis. Sci..

[41] Hughes S., Damato B.E., Giddings I., Hiscott P.S., Humphreys J., Houlston R.S. (2005). Microarray comparative genomic hybridisation analysis of intraocular uveal melanomas identifies distinctive imbalances associated with loss of chromosome 3. Br. J. Cancer.

[42] Narasimhaiah D., Legrand C., Damotte D., Remark R., Munda M., De Potter P., Coulie P.G., Vikkula M., Godfraind C. (2019). DNA alteration-based classification of uveal melanoma gives better prognostic stratification than immune infiltration, which has a neutral effect in high-risk group. Cancer Med..

[43] Robertson A.G., Shih J., Yau C., Gibb E.A., Oba J., Mungall K.L., Hess J.M., Uzunangelov V., Walter V., Danilova L. (2017). Integrative Analysis Identifies Four Molecular and Clinical Subsets in Uveal Melanoma. Cancer Cell.

[44] Minca E.C., Tubbs R.R., Portier B.P., Wang Z., Lanigan C., Aronow M.E., Triozzi P.L., Singh A., Cook J.R., Saunthararajah Y. (2014). Genomic microarray analysis on formalin-fixed paraffin-embedded material for uveal melanoma prognostication. Cancer Genet..

[45] Trolet J., Hupe P., Huon I., Lebigot I., Decraene C., Delattre O., Sastre-Garau X., Saule S., Thiery J.P., Plancher C. (2009). Genomic profiling and identification of high-risk uveal melanoma by array CGH analysis of primary tumors and liver metastases. Investig. Ophthalmol. Vis. Sci..

[46] Onken M.D., Worley L.A., Harbour J.W. (2008). A metastasis modifier locus on human chromosome 8p in uveal melanoma identified by integrative genomic analysis. Clin. Cancer Res..

[47] Mensink H.W., Kilic E., Vaarwater J., Douben H., Paridaens D., de Klein A. (2008). Molecular cytogenetic analysis of archival uveal melanoma with known clinical outcome. Cancer Genet. Cytogenet..

[48] Smit K.N., van Poppelen N.M., Vaarwater J., Verdijk R., van Marion R., Kalirai H., Coupland S.E., Thornton S., Farquhar N., Dubbink H.J. (2018). Combined mutation and copy-number variation detection by targeted next-generation sequencing in uveal melanoma. Mod. Pathol..

[49] Radhakrishnan A., Badhrinarayanan N., Biswas J., Krishnakumar S. (2009). Analysis of chromosomal aberration (1, 3, and 8) and association of microRNAs in uveal melanoma. Mol. Vis..

[50] Maat W., Ly L.V., Jordanova E.S., de Wolff-Rouendaal D., Schalij-Delfos N.E., Jager M.J. (2008). Monosomy of chromosome 3 and an inflammatory phenotype occur together in uveal melanoma. Investig. Ophthalmol. Vis. Sci..

[51] Ewens K.G., Kanetsky P.A., Richards-Yutz J., Al-Dahmash S., De Luca M.C., Bianciotto C.G., Shields C.L., Ganguly A. (2013). Genomic profile of 320 uveal melanoma cases: Chromosome 8p-loss and metastatic outcome. Investig. Ophthalmol. Vis. Sci..

[52] Drabarek W., Yavuzyigitoglu S., Obulkasim A., van Riet J., Smit K.N., van Poppelen N.M., Vaarwater J., Brands T., Eussen B., Verdijk R.M. (2019). Multi-Modality Analysis Improves Survival Prediction in Enucleated Uveal Melanoma Patients. Investig. Ophthalmol. Vis. Sci..

[53] Naus N.C., Verhoeven A.C., van Drunen E., Slater R., Mooy C.M., Paridaens D.A., Luyten G.P., de Klein A. (2002). Detection of genetic prognostic markers in uveal melanoma biopsies using fluorescence in situ hybridization. Clin. Cancer Res..

[54] Mudhar H.S., Doherty R.E., Salvi S.M., Currie Z.I., Tan J.H., Sisley K. (2019). Genetic Profiling of Primary Orbital Melanoma: An Analysis of 6 Cases with Clinicopathologic Correlation. Ophthalmology.

[55] Fang Y., Wang X., Dusza S., Jhanwar S., Abramson D., Busam K.J. (2012). Use of fluorescence in situ hybridization to distinguish metastatic uveal from cutaneous melanoma. Int. J. Surg. Pathol..

[56] Tura A., Merz H., Reinsberg M., Luke M., Jager M.J., Grisanti S., Luke J. (2016). Analysis of monosomy-3 in immunomagnetically isolated circulating melanoma cells in uveal melanoma patients. Pigment Cell Melanoma Res..

[57] Hamadeh F., Medina C.A., Singh A.D., Reynolds J.P., Biscotti C.V. (2016). Uveal melanoma: An analysis of cellular features and comparison to monosomy 3 status. Diagn. Cytopathol..

[58] Sandinha M.T., Farquharson M.A., Roberts F. (2004). Identification of monosomy 3 in choroidal melanoma by chromosome in situ hybridisation. Br. J. Ophthalmol..

[59] Klufas M.A., Richter E., Itty S., Moreno C., McCannel C.A., McCannel T.A. (2017). Comparison of Gene Expression Profiling and Chromosome 3 Analysis by Fluorescent in situ Hybridization and Multiplex Ligation Probe Amplification in Fine-Needle Aspiration Biopsy Specimens of Uveal Melanoma. Ocul. Oncol. Pathol..

[60] van den Bosch T., Vaarwater J., Verdijk R., Muller K., Kilic E., Paridaens D., de Klein A., Naus N. (2015). Risk factors associated with secondary enucleation after fractionated stereotactic radiotherapy in uveal melanoma. Acta Ophthalmol..

[61] Vaarwater J., van den Bosch T., Mensink H.W., van Kempen C., Verdijk R.M., Naus N.C., Paridaens D., Bruggenwirth H.T., Kilic E., de Klein A. (2012). Multiplex ligation-dependent probe amplification equals fluorescence in-situ hybridization for the identification of patients at risk for metastatic disease in uveal melanoma. Melanoma Res..

[62] Schoenfield L., Pettay J., Tubbs R.R., Singh A.D. (2009). Variation of monosomy 3 status within uveal melanoma. Arch. Pathol. Lab. Med..

[63] Coupland S.E., Campbell I., Damato B. (2008). Routes of extraocular extension of uveal melanoma: Risk factors and influence on survival probability. Ophthalmology.

[64] Tschentscher F., Prescher G., Zeschnigk M., Horsthemke B., Lohmann D.R. (2000). Identification of chromosomes 3, 6, and 8 aberrations in uveal melanoma by microsatellite analysis in comparison to comparative genomic hybridization. Cancer Genet. Cytogenet..

[65] Sandinha T., Farquharson M., McKay I., Roberts F. (2006). Correlation of heterogeneity for chromosome 3 copy number with cell type in choroidal melanoma of mixed-cell type. Investig. Ophthalmol. Vis. Sci..

[66] Singh A.D., Aronow M.E., Sun Y., Bebek G., Saunthararajah Y., Schoenfield L.R., Biscotti C.V., Tubbs R.R., Triozzi P.L., Eng C. (2012). Chromosome 3 status in uveal melanoma: A comparison of fluorescence in situ hybridization and single-nucleotide polymorphism array. Investig. Ophthalmol. Vis. Sci..

[67] Bonaldi L., Midena E., Filippi B., Tebaldi E., Marcato R., Parrozzani R., Amadori A. (2008). FISH analysis of chromosomes 3 and 6 on fine needle aspiration biopsy samples identifies distinct subgroups of uveal melanomas. J. Cancer Res. Clin. Oncol..

[68] Kilic E., Naus N.C., van Gils W., Klaver C.C., van Til M.E., Verbiest M.M., Stijnen T., Mooy C.M., Paridaens D., Beverloo H.B. (2005). Concurrent loss of chromosome arm 1p and chromosome 3 predicts a decreased disease-free survival in uveal melanoma patients. Investig. Ophthalmol. Vis. Sci..

[69] Dogrusoz M., Brouwer N.J., de Geus S.J.R., Ly L.V., Bohringer S., van Duinen S.G., Kroes W.G.M., van der Velden P.A., Haasnoot G.W., Marinkovic M. (2020). Prognostic Factors Five Years After Enucleation for Uveal Melanoma. Investig. Ophthalmol. Vis. Sci..

[70] Cassoux N., Rodrigues M.J., Plancher C., Asselain B., Levy-Gabriel C., Lumbroso-Le Rouic L., Piperno-Neumann S., Dendale R., Sastre X., Desjardins L. (2014). Genome-wide profiling is a clinically relevant and affordable prognostic test in posterior uveal melanoma. Br. J. Ophthalmol..

[71] Bronkhorst I.H., Maat W., Jordanova E.S., Kroes W.G., Schalij-Delfos N.E., Luyten G.P., Jager M.J. (2011). Effect of heterogeneous distribution of monosomy 3 on prognosis in uveal melanoma. Arch. Pathol. Lab. Med..

[72] Sisley K., Tattersall N., Dyson M., Smith K., Mudhar H.S., Rennie I.G. (2006). Multiplex fluorescence in situ hybridization identifies novel rearrangements of chromosomes 6, 15, and 18 in primary uveal melanoma. Exp. Eye Res..

[73] McCannel T.A., Reddy S., Burgess B.L., Auerbach M. (2010). Association of positive dual-modality positron emission tomography/computed tomography imaging of primary choroidal melanoma with chromosome 3 loss and tumor size. Retina.

[74] Dono M., Angelini G., Cecconi M., Amaro A., Esposito A.I., Mirisola V., Maric I., Lanza F., Nasciuti F., Viaggi S. (2014). Mutation frequencies of GNAQ, GNA11, BAP1, SF3B1, EIF1AX and TERT in uveal melanoma: Detection of an activating mutation in the TERT gene promoter in a single case of uveal melanoma. Br. J. Cancer.

[75] Glasgow B.J., McCannel T.A. (2018). Correlation of Immunocytochemistry of BRCA1-associated Protein-1 (BAP1) with Other Prognostic Markers in Uveal Melanoma. Am. J. Ophthalmol..

[76] Yavuzyigitoglu S., Mensink H.W., Smit K.N., Vaarwater J., Verdijk R.M., Beverloo B., Bruggenwirth H.T., van Marion R., Dubbink H.J., Paridaens D. (2016). Metastatic Disease in Polyploid Uveal Melanoma Patients Is Associated with BAP1 Mutations. Investig. Ophthalmol. Vis. Sci..

[77] Singh A.D., Tubbs R., Biscotti C., Schoenfield L., Trizzoi P. (2009). Chromosomal 3 and 8 status within hepatic metastasis of uveal melanoma. Arch. Pathol. Lab. Med..

[78] Maat W., Jordanova E.S., van Zelderen-Bhola S.L., Barthen E.R., Wessels H.W., Schalij-Delfos N.E., Jager M.J. (2007). The heterogeneous distribution of monosomy 3 in uveal melanomas: Implications for prognostication based on fine-needle aspiration biopsies. Arch. Pathol. Lab. Med..

[79] Tschentscher F., Prescher G., Horsman D.E., White V.A., Rieder H., Anastassiou G., Schilling H., Bornfeld N., Bartz-Schmidt K.U., Horsthemke B. (2001). Partial deletions of the long and short arm of chromosome 3 point to two tumor suppressor genes in uveal melanoma. Cancer Res..

[80] Young T.A., Burgess B.L., Rao N.P., Glasgow B.J., Straatsma B.R. (2008). Transscleral fine-needle aspiration biopsy of macular choroidal melanoma. Am. J. Ophthalmol..

[81] Sipos E., Hegyi K., Treszl A., Steiber Z., Mehes G., Dobos N., Fodor K., Olah G., Szekvolgyi L., Schally A.V. (2017). Concurrence of chromosome 3 and 4 aberrations in human uveal melanoma. Oncol. Rep..

[82] Hammond D.W., Al-Shammari N.S., Danson S., Jacques R., Rennie I.G., Sisley K. (2015). High-Resolution Array CGH Analysis Identifies Regional Deletions and Amplifications of Chromosome 8 in Uveal Melanoma. Investig. Ophthalmol. Vis. Sci..

[83] Midena E., Bonaldi L., Parrozzani R., Radin P.P., Boccassini B., Vujosevic S. (2008). In vivo monosomy 3 detection of posterior uveal melanoma: 3-year follow-up. Graefes Arch. Clin. Exp. Ophthalmol..

[84] Erol N., Oner U., Artan S., Isiksoy S., Yurdakul S. (2004). Chromosomal abnormalities, p53 and Bcl-2 expression and clinical outcome in choroidal melanoma. Melanoma Res..

[85] Abdel-Rahman M.H., Christopher B.N., Faramawi M.F., Said-Ahmed K., Cole C., McFaddin A., Ray-Chaudhury A., Heerema N., Davidorf F.H. (2011). Frequency, molecular pathology and potential clinical significance of partial chromosome 3 aberrations in uveal melanoma. Mod. Pathol..

[86] van Beek J.G., Koopmans A.E., Vaarwater J., de Rooi J.J., Paridaens D., Naus N.C., de Klein A., Verdijk R.M., Kilic E. (2014). The prognostic value of extraocular extension in relation to monosomy 3 and gain of chromosome 8q in uveal melanoma. Investig. Ophthalmol. Vis. Sci..

[87] Lee C.S., Lee J., Choi J.J., Yang W.I., Yoon J.S., Lee S.Y., Lee S.C. (2011). Cytogenetics and prognosis for uveal melanoma in Korean patients. Acta Ophthalmol..

[88] Damato B., Duke C., Coupland S.E., Hiscott P., Smith P.A., Campbell I., Douglas A., Howard P. (2007). Cytogenetics of uveal melanoma: A 7-year clinical experience. Ophthalmology.

[89] Matet A., Ait Rais K., Malaise D., Angi M., Dendale R., Tick S., Lumbroso-Le Rouic L., Levy-Gabriel C., Rodrigues M., Pierron G. (2019). Comparative Cytogenetic Abnormalities in Paired Choroidal Melanoma Samples Obtained Before and After Proton Beam Irradiation by Transscleral Fine-Needle Aspiration Biopsy and Endoresection. Cancers.

[90] van Poppelen N.M., Vaarwater J., Mudhar H.S., Sisley K., Rennie I.G., Rundle P., Brands T., van den Bosch Q.C.C., Mensink H.W., de Klein A. (2018). Genetic Background of Iris Melanomas and Iris Melanocytic Tumors of Uncertain Malignant Potential. Ophthalmology.

[91] Tura A., Thieme C., Brosig A., Merz H., Ranjbar M., Vardanyan S., Zuo H., Maassen T., Kakkassery V., Grisanti S. (2020). Lower Levels of Adiponectin and Its Receptor Adipor1 in the Uveal Melanomas with Monosomy-3. Investig. Ophthalmol. Vis. Sci..

[92] Versluis M., de Lange M.J., van Pelt S.I., Ruivenkamp C.A., Kroes W.G., Cao J., Jager M.J., Luyten G.P., van der Velden P.A. (2015). Digital PCR validates 8q dosage as prognostic tool in uveal melanoma. PLoS ONE.

[93] van Essen T.H., van Pelt S.I., Versluis M., Bronkhorst I.H., van Duinen S.G., Marinkovic M., Kroes W.G., Ruivenkamp C.A., Shukla S., de Klein A. (2014). Prognostic parameters in uveal melanoma and their association with BAP1 expression. Br. J. Ophthalmol..

[94] Lake S.L., Damato B.E., Kalirai H., Dodson A.R., Taktak A.F., Lloyd B.H., Coupland S.E. (2013). Single nucleotide polymorphism array analysis of uveal melanomas reveals that amplification of CNKSR3 is correlated with improved patient survival. Am. J. Pathol..

[95] Mensink H.W., Vaarwater J., Kilic E., Naus N.C., Mooy N., Luyten G., Bruggenwirth H.T., Paridaens D., de Klein A. (2009). Chromosome 3 intratumor heterogeneity in uveal melanoma. Investig. Ophthalmol. Vis. Sci..

[96] Petrausch U., Martus P., Tonnies H., Bechrakis N.E., Lenze D., Wansel S., Hummel M., Bornfeld N., Thiel E., Foerster M.H. (2008). Significance of gene expression analysis in uveal melanoma in comparison to standard risk factors for risk assessment of subsequent metastases. Eye.

[97] Marathe O.S., Wu J., Lee S.P., Yu F., Burgess B.L., Leu M., Straatsma B.R., McCannel T.A. (2011). Ocular response of choroidal melanoma with monosomy 3 versus disomy 3 after iodine-125 brachytherapy. Int. J. Radiat. Oncol. Biol. Phys..

[98] McCannel T.A., Burgess B.L., Rao N.P., Nelson S.F., Straatsma B.R. (2010). Identification of candidate tumor oncogenes by integrative molecular analysis of choroidal melanoma fine-needle aspiration biopsy specimens. Arch. Ophthalmol..

[99] Dogrusoz M., Bagger M., van Duinen S.G., Kroes W.G., Ruivenkamp C.A., Bohringer S., Andersen K.K., Luyten G.P., Kiilgaard J.F., Jager M.J. (2017). The Prognostic Value of AJCC Staging in Uveal Melanoma Is Enhanced by Adding Chromosome 3 and 8q Status. Investig. Ophthalmol. Vis. Sci..

[100] Zimpfer A., Schneider B., Blanck O., Riedel K., Zhivov A., Jonigk D., Erbersdobler A., Junemann A., Andratschke N., Hildebrandt G. (2019). Pathologic Features of Tumor Activity and Stability in Uveal Melanoma Specimens after Fractionated CyberKnife Radiosurgery. Pathol. Oncol. Res..

[101] Shields C.L., Say E.A.T., Hasanreisoglu M., Saktanasate J., Lawson B.M., Landy J.E., Badami A.U., Sivalingam M.D., Mashayekhi A., Shields J.A. (2017). Cytogenetic Abnormalities in Uveal Melanoma Based on Tumor Features and Size in 1059 Patients: The 2016 W. Richard Green Lecture. Ophthalmology.

[102] Salvi S.M., Aziz H.A., Dar S., Singh N., Hayden-Loreck B., Singh A.D. (2017). Uveal Melanoma Regression after Brachytherapy: Relationship with Chromosome 3 Monosomy Status. Ocul. Oncol. Pathol..

[103] Young T.A., Rao N.P., Glasgow B.J., Moral J.N., Straatsma B.R. (2007). Fluorescent in situ hybridization for monosomy 3 via 30-gauge fine-needle aspiration biopsy of choroidal melanoma in vivo. Ophthalmology.

[104] McCarthy C., Kalirai H., Lake S.L., Dodson A., Damato B.E., Coupland S.E. (2016). Insights into genetic alterations of liver metastases from uveal melanoma. Pigment Cell Melanoma Res..

[105] van den Bosch T., Koopmans A.E., Vaarwater J., van den Berg M., de Klein A., Verdijk R.M. (2013). Chemokine receptor CCR7 expression predicts poor outcome in uveal melanoma and relates to liver metastasis whereas expression of CXCR4 is not of clinical relevance. Investig. Ophthalmol. Vis. Sci..

[106] Ramtohul T., Ait Rais K., Gardrat S., Barnhill R., Roman-Roman S., Cassoux N., Rodrigues M., Mariani P., De Koning L., Pierron G. (2021). Prognostic Implications of MRI Melanin Quantification and Cytogenetic Abnormalities in Liver Metastases of Uveal Melanoma. Cancers.

[107] van den Bosch T., van Beek J.G., Vaarwater J., Verdijk R.M., Naus N.C., Paridaens D., de Klein A., Kilic E. (2012). Higher percentage of FISH-determined monosomy 3 and 8q amplification in uveal melanoma cells relate to poor patient prognosis. Investig. Ophthalmol. Vis. Sci..

[108] Patel K.A., Edmondson N.D., Talbot F., Parsons M.A., Rennie I.G., Sisley K. (2001). Prediction of prognosis in patients with uveal melanoma using fluorescence in situ hybridisation. Br. J. Ophthalmol..

[109] Alkatan H.M., Al Qahtani A.A., Maktabi A.M. (2020). Enucleated globes with choroidal melanoma: A retrospective histopathological study and correlation with cytogenetic profile in 2 eye centers. Ann. Med. Surg..

[110] Rodrigues M., Ait Rais K., Salviat F., Algret N., Simaga F., Barnhill R., Gardrat S., Servois V., Mariani P., Piperno-Neumann S. (2020). Association of Partial Chromosome 3 Deletion in Uveal Melanomas with Metastasis-Free Survival. JAMA Ophthalmol..

[111] Mensink H.W., Vaarwater J., de Keizer R.J., de Wolff-Rouendaal D., Mooy C.M., de Klein A., Paridaens D. (2011). Chromosomal aberrations in iris melanomas. Br. J. Ophthalmol..

[112] Cross N.A., Ganesh A., Parpia M., Murray A.K., Rennie I.G., Sisley K. (2006). Multiple locations on chromosome 3 are the targets of specific deletions in uveal melanoma. Eye.

[113] Lake S.L., Coupland S.E., Taktak A.F., Damato B.E. (2010). Whole-genome microarray detects deletions and loss of heterozygosity of chromosome 3 occurring exclusively in metastasizing uveal melanoma. Investig. Ophthalmol. Vis. Sci..

[114] Chang M.Y., Rao N.P., Burgess B.L., Johnson L., McCannel T.A. (2013). Heterogeneity of monosomy 3 in fine needle aspiration biopsy of choroidal melanoma. Mol. Vis..

[115] Ehlers J.P., Worley L., Onken M.D., Harbour J.W. (2008). Integrative genomic analysis of aneuploidy in uveal melanoma. Clin. Cancer Res..

[116] Young T.A., Burgess B.L., Rao N.P., Gorin M.B., Straatsma B.R. (2007). High-density genome array is superior to fluorescence in-situ hybridization analysis of monosomy 3 in choroidal melanoma fine needle aspiration biopsy. Mol. Vis..

[117] Field M.G., Decatur C.L., Kurtenbach S., Gezgin G., van der Velden P.A., Jager M.J., Kozak K.N., Harbour J.W. (2016). PRAME as an Independent Biomarker for Metastasis in Uveal Melanoma. Clin. Cancer Res..

[118] van Engen-van Grunsven A.C., Baar M.P., Pfundt R., Rijntjes J., Kusters-Vandevelde H.V., Delbecq A.L., Keunen J.E., Klevering J.B., Wesseling P., Blokx W.A. (2015). Whole-genome copy-number analysis identifies new leads for chromosomal aberrations involved in the oncogenesis and metastastic behavior of uveal melanomas. Melanoma Res..

[119] Bagger M., Andersen M.T., Heegaard S., Andersen M.K., Kiilgaard J.F. (2015). Transvitreal Retinochoroidal Biopsy Provides a Representative Sample From Choroidal Melanoma for Detection of Chromosome 3 Aberrations. Investig. Ophthalmol. Vis. Sci..

[120] Abdel-Rahman M.H., Craig E.L., Davidorf F.H., Eng C. (2005). Expression of vascular endothelial growth factor in uveal melanoma is independent of 6p21-region copy number. Clin. Cancer Res..

[121] Griewank K.G., Westekemper H., Murali R., Mach M., Schilling B., Wiesner T., Schimming T., Livingstone E., Sucker A., Grabellus F. (2013). Conjunctival melanomas harbor BRAF and NRAS mutations and copy number changes similar to cutaneous and mucosal melanomas. Clin. Cancer Res..

[122] Moore M.W., Gasparini R. (2011). FISH as an effective diagnostic tool for the management of challenging melanocytic lesions. Diagn. Pathol..

[123] Diaz A., Valera A., Carrera C., Hakim S., Aguilera P., Garcia A., Palou J., Puig S., Malvehy J., Alos L. (2011). Pigmented spindle cell nevus: Clues for differentiating it from spindle cell malignant melanoma. A comprehensive survey including clinicopathologic, immunohistochemical, and FISH studies. Am. J. Surg. Pathol..

[124] Fang Y., Dusza S., Jhanwar S., Busam K.J. (2012). Fluorescence in situ hybridization (FISH) analysis of melanocytic nevi and melanomas: Sensitivity, specificity, and lack of association with sentinel node status. Int. J. Surg. Pathol..

[125] Tetzlaff M.T., Wang W.L., Milless T.L., Curry J.L., Torres-Cabala C.A., McLemore M.S., Ivan D., Bassett R.L., Prieto V.G. (2013). Ambiguous melanocytic tumors in a tertiary referral center: The contribution of fluorescence in situ hybridization (FISH) to conventional histopathologic and immunophenotypic analyses. Am. J. Surg. Pathol..

[126] Alomari A.K., Harms P.W., Andea A.A., Warren S.J. (2023). MAP2K1-mutated melanocytic tumors have reproducible histopathologic features and share similarities with melanocytic tumors with BRAF V600E mutations. J. Cutan. Pathol..

[127] Raghavan S.S., Peternel S., Mully T.W., North J.P., Pincus L.B., LeBoit P.E., McCalmont T.H., Bastian B.C., Yeh I. (2020). Spitz melanoma is a distinct subset of spitzoid melanoma. Mod. Pathol..

[128] Chatzopoulos K., Syrnioti A., Linos K. (2024). Spitz Melanocytic Tumors: A Fascinating 75-Year Journey. Genes.

[129] Bastian B.C., Wesselmann U., Pinkel D., Leboit P.E. (1999). Molecular cytogenetic analysis of Spitz nevi shows clear differences to melanoma. J. Investig. Dermatol..

[130] Pappo A.S., McPherson V., Pan H., Wang F., Wang L., Wright T., Hussong M., Hawkins D., Kaste S.C., Davidoff A.M. (2021). A prospective, comprehensive registry that integrates the molecular analysis of pediatric and adolescent melanocytic lesions. Cancer.

[131] DeMarchis E.H., Swetter S.M., Jennings C.D., Kim J. (2014). Fluorescence in situ hybridization analysis of atypical melanocytic proliferations and melanoma in young patients. Pediatr. Dermatol..

[132] Gammon B., Beilfuss B., Guitart J., Gerami P. (2012). Enhanced detection of spitzoid melanomas using fluorescence in situ hybridization with 9p21 as an adjunctive probe. Am. J. Surg. Pathol..

[133] Gerami P., Li G., Pouryazdanparast P., Blondin B., Beilfuss B., Slenk C., Du J., Guitart J., Jewell S., Pestova K. (2012). A highly specific and discriminatory FISH assay for distinguishing between benign and malignant melanocytic neoplasms. Am. J. Surg. Pathol..

[134] Gerami P., Scolyer R.A., Xu X., Elder D.E., Abraham R.M., Fullen D., Prieto V.G., Leboit P.E., Barnhill R.L., Cooper C. (2013). Risk assessment for atypical spitzoid melanocytic neoplasms using FISH to identify chromosomal copy number aberrations. Am. J. Surg. Pathol..

[135] Bastian B.C., Olshen A.B., LeBoit P.E., Pinkel D. (2003). Classifying melanocytic tumors based on DNA copy number changes. Am. J. Pathol..

[136] Yeh I., Busam K.J. (2022). Spitz melanocytic tumours—A review. Histopathology.

[137] Cheng T.W., Ahern M.C., Giubellino A. (2022). The Spectrum of Spitz Melanocytic Lesions: From Morphologic Diagnosis to Molecular Classification. Front. Oncol..

[138] Turner J., Couts K., Sheren J., Saichaemchan S., Ariyawutyakorn W., Avolio I., Cabral E., Glogowska M., Amato C., Robinson S. (2017). Kinase gene fusions in defined subsets of melanoma. Pigment Cell Melanoma Res..

[139] Delsupehe L., Steelandt T., Lemahieu J., Volders P.J., Geerdens E., Berden S., Daniels A., Froyen G., Maes B. (2024). Novel gene fusion discovery in Spitz tumours and its relevance in diagnostics. Virchows Arch..

[140] Gassenmaier M., Soltanpour N., Held L., Metzler G., Yazdi A.S., Brecht I.B., Schneider D.T., Stadler R., Garbe C., Bauer J. (2022). Diagnostic and prognostic classification of atypical spitzoid tumours based on histology and genomic aberrations: A prospective cohort study with long-term follow-up. Eur. J. Cancer.

[141] Wiesner T., Murali R., Fried I., Cerroni L., Busam K., Kutzner H., Bastian B.C. (2012). A distinct subset of atypical Spitz tumors is characterized by BRAF mutation and loss of BAP1 expression. Am. J. Surg. Pathol..

[142] Yeh I., Mully T.W., Wiesner T., Vemula S.S., Mirza S.A., Sparatta A.J., McCalmont T.H., Bastian B.C., LeBoit P.E. (2014). Ambiguous melanocytic tumors with loss of 3p21. Am. J. Surg. Pathol..

[143] Cabrera R., Recule F. (2018). Unusual Clinical Presentations of Malignant Melanoma: A Review of Clinical and Histologic Features with Special Emphasis on Dermatoscopic Findings. Am. J. Clin. Dermatol..

[144] Vergier B., Laharanne E., Prochazkova-Carlotti M., de la Fouchardiere A., Merlio J.P., Kadlub N., Avril M.F., Bodemer C., Lacoste C., Boralevi F. (2016). Proliferative Nodules vs Melanoma Arising in Giant Congenital Melanocytic Nevi During Childhood. JAMA Dermatol..

[145] Clemente C., Bettio D., Venci A., Scopsi L., Rao S., Ferrari A., Piris A., Mihm M.C. (2009). A fluorescence in situ hybridization (FISH) procedure to assist in differentiating benign from malignant melanocytic lesions. Pathologica.

[146] Gerami P., Beilfuss B., Haghighat Z., Fang Y., Jhanwar S., Busam K.J. (2011). Fluorescence in situ hybridization as an ancillary method for the distinction of desmoplastic melanomas from sclerosing melanocytic nevi. J. Cutan. Pathol..

[147] Maize J.C., McCalmont T.H., Carlson J.A., Busam K.J., Kutzner H., Bastian B.C. (2005). Genomic analysis of blue nevi and related dermal melanocytic proliferations. Am. J. Surg. Pathol..

[148] van Dijk M., Sprenger S., Rombout P., Marres H., Kaanders J., Jeuken J., Ruiter D. (2003). Distinct chromosomal aberrations in sinonasal mucosal melanoma as detected by comparative genomic hybridization. Genes Chromosomes Cancer.

[149] Chlopek M., Lasota J., Thompson L.D.R., Szczepaniak M., Kuzniacka A., Hincza K., Kubicka K., Kaczorowski M., Newford M., Liu Y. (2022). Alterations in key signaling pathways in sinonasal tract melanoma. A molecular genetics and immunohistochemical study of 90 cases and comprehensive review of the literature. Mod. Pathol..

[150] Furney S.J., Turajlic S., Stamp G., Nohadani M., Carlisle A., Thomas J.M., Hayes A., Strauss D., Gore M., van den Oord J. (2013). Genome sequencing of mucosal melanomas reveals that they are driven by distinct mechanisms from cutaneous melanoma. J. Pathol..

[151] Newell F., Kong Y., Wilmott J.S., Johansson P.A., Ferguson P.M., Cui C., Li Z., Kazakoff S.H., Burke H., Dodds T.J. (2019). Whole-genome landscape of mucosal melanoma reveals diverse drivers and therapeutic targets. Nat. Commun..

[152] Broit N., Johansson P.A., Rodgers C.B., Walpole S.T., Newell F., Hayward N.K., Pritchard A.L. (2021). Meta-Analysis and Systematic Review of the Genomics of Mucosal Melanoma. Mol. Cancer Res..

[153] Hodi F.S., Corless C.L., Giobbie-Hurder A., Fletcher J.A., Zhu M., Marino-Enriquez A., Friedlander P., Gonzalez R., Weber J.S., Gajewski T.F. (2013). Imatinib for melanomas harboring mutationally activated or amplified KIT arising on mucosal, acral, and chronically sun-damaged skin. J. Clin. Oncol..

[154] Bastian B.C., Kashani-Sabet M., Hamm H., Godfrey T., Moore D.H., Brocker E.B., LeBoit P.E., Pinkel D. (2000). Gene amplifications characterize acral melanoma and permit the detection of occult tumor cells in the surrounding skin. Cancer Res..

[155] North J.P., Vetto J.T., Murali R., White K.P., White C.R., Bastian B.C. (2011). Assessment of copy number status of chromosomes 6 and 11 by FISH provides independent prognostic information in primary melanoma. Am. J. Surg. Pathol..

[156] Gandolfi G., Longo C., Moscarella E., Zalaudek I., Sancisi V., Raucci M., Manzotti G., Gugnoni M., Piana S., Argenziano G. (2016). The extent of whole-genome copy number alterations predicts aggressive features in primary melanomas. Pigment Cell Melanoma Res..

[157] Alomari A.K., Miedema J.R., Carter M.D., Harms P.W., Lowe L., Durham A.B., Fullen D.R., Patel R.M., Hristov A.C., Chan M.P. (2020). DNA copy number changes correlate with clinical behavior in melanocytic neoplasms: Proposal of an algorithmic approach. Mod. Pathol..

[158] Balazs M., Adam Z., Treszl A., Begany A., Hunyadi J., Adany R. (2001). Chromosomal imbalances in primary and metastatic melanomas revealed by comparative genomic hybridization. Cytometry.

[159] Stephens P.J., Greenman C.D., Fu B., Yang F., Bignell G.R., Mudie L.J., Pleasance E.D., Lau K.W., Beare D., Stebbings L.A. (2011). Massive genomic rearrangement acquired in a single catastrophic event during cancer development. Cell.

[160] Hirsch D., Kemmerling R., Davis S., Camps J., Meltzer P.S., Ried T., Gaiser T. (2013). Chromothripsis and focal copy number alterations determine poor outcome in malignant melanoma. Cancer Res..

[161] Cortes-Ciriano I., Lee J.J., Xi R., Jain D., Jung Y.L., Yang L., Gordenin D., Klimczak L.J., Zhang C.Z., Pellman D.S. (2020). Comprehensive analysis of chromothripsis in 2,658 human cancers using whole-genome sequencing. Nat. Genet..

[162] Yeh I., Botton T., Talevich E., Shain A.H., Sparatta A.J., de la Fouchardiere A., Mully T.W., North J.P., Garrido M.C., Gagnon A. (2015). Activating MET kinase rearrangements in melanoma and Spitz tumours. Nat. Commun..

[163] Quan V.L., Panah E., Zhang B., Shi K., Mohan L.S., Gerami P. (2019). The role of gene fusions in melanocytic neoplasms. J. Cutan. Pathol..

[164] de la Fouchardiere A., Tee M.K., Peternel S., Valdebran M., Pissaloux D., Tirode F., Busam K.J., LeBoit P.E., McCalmont T.H., Bastian B.C. (2021). Fusion partners of NTRK3 affect subcellular localization of the fusion kinase and cytomorphology of melanocytes. Mod. Pathol..

[165] Roy S.F., Milante R., Pissaloux D., Tirode F., Bastian B.C., Fouchardiere A., Yeh I. (2023). Spectrum of Melanocytic Tumors Harboring BRAF Gene Fusions: 58 Cases with Histomorphologic and Genetic Correlations. Mod. Pathol..

[166] Shorokhova M., Nikolsky N., Grinchuk T. (2021). Chromothripsis-Explosion in Genetic Science. Cells.

[167] Luijten M.N.H., Lee J.X.T., Crasta K.C. (2018). Mutational game changer: Chromothripsis and its emerging relevance to cancer. Mutat. Res. Rev. Mutat. Res..

[168] Yeh I. (2022). Update on classification of melanocytic tumors and the role of immunohistochemistry and molecular techniques. Semin. Diagn. Pathol..

[169] Poetsch M., Woenckhaus C., Dittberner T., Pambor M., Lorenz G., Herrmann F.H. (1999). Significance of the small subtelomeric area of chromosome 1 (1p36.3) in the progression of malignant melanoma: FISH deletion screening with YAC DNA probes. Virchows Arch..

[170] Wang J., Hirose H., Du G., Chong K., Kiyohara E., Witz I.P., Hoon D.S.B. (2017). P-REX1 amplification promotes progression of cutaneous melanoma via the PAK1/P38/MMP-2 pathway. Cancer Lett..

[171] Greshock J., Nathanson K., Medina A., Ward M.R., Herlyn M., Weber B.L., Zaks T.Z. (2009). Distinct patterns of DNA copy number alterations associate with BRAF mutations in melanomas and melanoma-derived cell lines. Genes Chromosomes Cancer.

[172] Stagni C., Zamuner C., Elefanti L., Zanin T., Bianco P.D., Sommariva A., Fabozzi A., Pigozzo J., Mocellin S., Montesco M.C. (2018). BRAF Gene Copy Number and Mutant Allele Frequency Correlate with Time to Progression in Metastatic Melanoma Patients Treated with MAPK Inhibitors. Mol. Cancer Ther..

[173] Vizkeleti L., Ecsedi S., Rakosy Z., Orosz A., Lazar V., Emri G., Koroknai V., Kiss T., Adany R., Balazs M. (2012). The role of CCND1 alterations during the progression of cutaneous malignant melanoma. Tumour Biol..

[174] Gerami P., Jewell S.S., Pouryazdanparast P., Wayne J.D., Haghighat Z., Busam K.J., Rademaker A., Morrison L. (2011). Copy number gains in 11q13 and 8q24 [corrected] are highly linked to prognosis in cutaneous malignant melanoma. J. Mol. Diagn..

[175] Kraehn G.M., Utikal J., Udart M., Greulich K.M., Bezold G., Kaskel P., Leiter U., Peter R.U. (2001). Extra c-myc oncogene copies in high risk cutaneous malignant melanoma and melanoma metastases. Br. J. Cancer.

[176] Pouryazdanparast P., Cowen D.P., Beilfuss B.A., Haghighat Z., Guitart J., Rademaker A., Gerami P. (2012). Distinctive clinical and histologic features in cutaneous melanoma with copy number gains in 8q24. Am. J. Surg. Pathol..

[177] Namiki T., Yanagawa S., Izumo T., Ishikawa M., Tachibana M., Kawakami Y., Yokozeki H., Nishioka K., Kaneko Y. (2005). Genomic alterations in primary cutaneous melanomas detected by metaphase comparative genomic hybridization with laser capture or manual microdissection: 6p gains may predict poor outcome. Cancer Genet. Cytogenet..

[178] Rakosy Z., Ecsedi S., Toth R., Vizkeleti L., Hernandez-Vargas H., Lazar V., Emri G., Szatmari I., Herceg Z., Adany R. (2013). Integrative genomics identifies gene signature associated with melanoma ulceration. PLoS ONE.

[179] Udart M., Utikal J., Krahn G.M., Peter R.U. (2001). Chromosome 7 aneusomy. A marker for metastatic melanoma? Expression of the epidermal growth factor receptor gene and chromosome 7 aneusomy in nevi, primary malignant melanomas and metastases. Neoplasia.

[180] Boone B., Jacobs K., Ferdinande L., Taildeman J., Lambert J., Peeters M., Bracke M., Pauwels P., Brochez L. (2011). EGFR in melanoma: Clinical significance and potential therapeutic target. J. Cutan. Pathol..

[181] Rakosy Z., Vizkeleti L., Ecsedi S., Voko Z., Begany A., Barok M., Krekk Z., Gallai M., Szentirmay Z., Adany R. (2007). EGFR gene copy number alterations in primary cutaneous malignant melanomas are associated with poor prognosis. Int. J. Cancer.

[182] De Semir D., Nosrati M., Bezrookove V., Dar A.A., Federman S., Bienvenu G., Venna S., Rangel J., Climent J., Meyer Tamguney T.M. (2012). Pleckstrin homology domain-interacting protein (PHIP) as a marker and mediator of melanoma metastasis. Proc. Natl. Acad. Sci. USA.

[183] Bezrookove V., De Semir D., Nosrati M., Tong S., Wu C., Thummala S., Dar A.A., Leong S.P.L., Cleaver J.E., Sagebiel R.W. (2014). Prognostic impact of PHIP copy number in melanoma: Linkage to ulceration. J. Investig. Dermatol..

[184] Bezrookove V., Nosrati M., Miller J.R., De Semir D., Dar A.A., Vosoughi E., Vaquero E., Sucker A., Lazar A.J., Gershenwald J.E. (2018). Role of Elevated PHIP Copy Number as a Prognostic and Progression Marker for Cutaneous Melanoma. Clin. Cancer Res..

[185] Papp O., Doma V., Gil J., Marko-Varga G., Karpati S., Timar J., Vizkeleti L. (2021). Organ Specific Copy Number Variations in Visceral Metastases of Human Melanoma. Cancers.

[186] Kabbarah O., Nogueira C., Feng B., Nazarian R.M., Bosenberg M., Wu M., Scott K.L., Kwong L.N., Xiao Y., Cordon-Cardo C. (2010). Integrative genome comparison of primary and metastatic melanomas. PLoS ONE.

[187] Yu S., Xu T., Dai J., Ma M., Tang H., Chi Z., Si L., Cui C., Sheng X., Kong Y. (2018). TERT copy gain predicts the outcome of high-dose interferon alpha-2b therapy in acral melanoma. OncoTargets Ther..

[188] Forschner A., Hilke F.J., Bonzheim I., Gschwind A., Demidov G., Amaral T., Ossowski S., Riess O., Schroeder C., Martus P. (2020). MDM2, MDM4 and EGFR Amplifications and Hyperprogression in Metastatic Acral and Mucosal Melanoma. Cancers.

[189] Yu J., Yan J., Guo Q., Chi Z., Tang B., Zheng B., Yu J., Yin T., Cheng Z., Wu X. (2019). Genetic Aberrations in the CDK4 Pathway Are Associated with Innate Resistance to PD-1 Blockade in Chinese Patients with Non-Cutaneous Melanoma. Clin. Cancer Res..

[190] Perottet J., Le Goff E., Legoupil D., Quere G., Schick U., Marcorelles P., Uguen A. (2020). PD-L1 Copy Number Variation Does Not Correlate with PD-L1 Expression or Response to Anti-PD-1 Immunotherapy In Patients with Advanced Melanomas. Appl. Immunohistochem. Mol. Morphol..

[191] Ramani N.S., Morani A.C., Zhang S. (2022). MET Gene High Copy Number (Amplification/Polysomy) Identified in Melanoma for Potential Targeted Therapy. Am. J. Clin. Pathol..

[192] Uguen A., Uguen M., Guibourg B., Talagas M., Marcorelles P., De Braekeleer M. (2018). The p16-Ki-67-HMB45 Immunohistochemistry Scoring System is Highly Concordant with the Fluorescent In Situ Hybridization Test to Differentiate Between Melanocytic Nevi and Melanomas. Appl. Immunohistochem. Mol. Morphol..

[193] Redon S., Guibourg B., Talagas M., Marcorelles P., Uguen A. (2018). A Diagnostic Algorithm Combining Immunohistochemistry and Molecular Cytogenetics to Diagnose Challenging Melanocytic Tumors. Appl. Immunohistochem. Mol. Morphol..

[194] Cho-Vega J.H. (2016). A diagnostic algorithm for atypical spitzoid tumors: Guidelines for immunohistochemical and molecular assessment. Mod. Pathol..

[195] Lezcano C., Jungbluth A.A., Busam K.J. (2020). Comparison of Immunohistochemistry for PRAME with Cytogenetic Test Results in the Evaluation of Challenging Melanocytic Tumors. Am. J. Surg. Pathol..

[196] Alomari A.K., Tharp A.W., Umphress B., Kowal R.P. (2021). The utility of PRAME immunohistochemistry in the evaluation of challenging melanocytic tumors. J. Cutan. Pathol..

[197] Thomas N.E., Edmiston S.N., Tsai Y.S., Parker J.S., Googe P.B., Busam K.J., Scott G.A., Zedek D.C., Parrish E.A., Hao H. (2019). Utility of TERT Promoter Mutations for Cutaneous Primary Melanoma Diagnosis. Am. J. Dermatopathol..

[198] Motaparthi K., Kim J., Andea A.A., Missall T.A., Novoa R.A., Vidal C.I., Fung M.A., Emanuel P.O. (2020). TERT and TERT promoter in melanocytic neoplasms: Current concepts in pathogenesis, diagnosis, and prognosis. J. Cutan. Pathol..

[199] Minca E.C., Al-Rohil R.N., Wang M., Harms P.W., Ko J.S., Collie A.M., Kovalyshyn I., Prieto V.G., Tetzlaff M.T., Billings S.D. (2016). Comparison between melanoma gene expression score and fluorescence in situ hybridization for the classification of melanocytic lesions. Mod. Pathol..

[200] Reimann J.D.R., Salim S., Velazquez E.F., Wang L., Williams K.M., Flejter W.L., Brooke L., Sunder S., Busam K.J. (2018). Comparison of melanoma gene expression score with histopathology, fluorescence in situ hybridization, and SNP array for the classification of melanocytic neoplasms. Mod. Pathol..

[201] Castillo S.A., Pham A.K., Dagrosa A.T., Yan S., Barton D.T., Lefferts J.A., Linos K. (2020). Concordance Analysis of the 23-Gene Expression Signature (myPath Melanoma) with Fluorescence In Situ Hybridization Assay and Single Nucleotide Polymorphism Array in the Analysis of Challenging Melanocytic Lesions: Results From an Academic Medical Center. Am. J. Dermatopathol..

[202] Clarke L.E., Pimentel J.D., Zalaznick H., Wang L., Busam K.J. (2017). Gene expression signature as an ancillary method in the diagnosis of desmoplastic melanoma. Hum. Pathol..

[203] Du R., Dong J., Jiang H., Qi M., Zhao Z. (2025). Comparative study of tools for copy number variation detection using next-generation sequencing data. Sci. Rep..

[204] Ferrara G., Misciali C., Brenn T., Cerroni L., Kazakov D.W., Perasole A., Russo R., Ricci R., Crisman G., Fanti P.A. (2013). The impact of molecular morphology techniques on the expert diagnosis in melanocytic skin neoplasms. Int. J. Surg. Pathol..

[205] Cloutier J.M., Wang M., Vemula S.S., Mirza S., Weier J., Aquino J.D., McCalmont T.H., LeBoit P.E., Bastian B.C., Yeh I. (2024). Amplification of Mutant NRAS in Melanocytic Tumors with Features of Spitz Tumors. Mod. Pathol..

