# Supplementary material for: Clinical Utility of Copy Number Abnormality Analysis in the Evaluation of Melanocytic Lesions for Diagnosis and Prognosis: An Evidence-Based Review from the Cancer Genomics Consortium Working Group for Melanocytic Lesions
Source: Genes (Basel). 2026 Mar 18;17(3):331. doi: 10.3390/genes17030331 (PMC13026022; doi:10.3390/genes17030331)
Supplement: Supplementary file 1 [file genes-17-00331-s001.zip › genes-4198361-supplementary.pdf]

Supplementary Figure S1: PRISMA 2020 flow diagram for systematic reviews including searches of databases. [36]

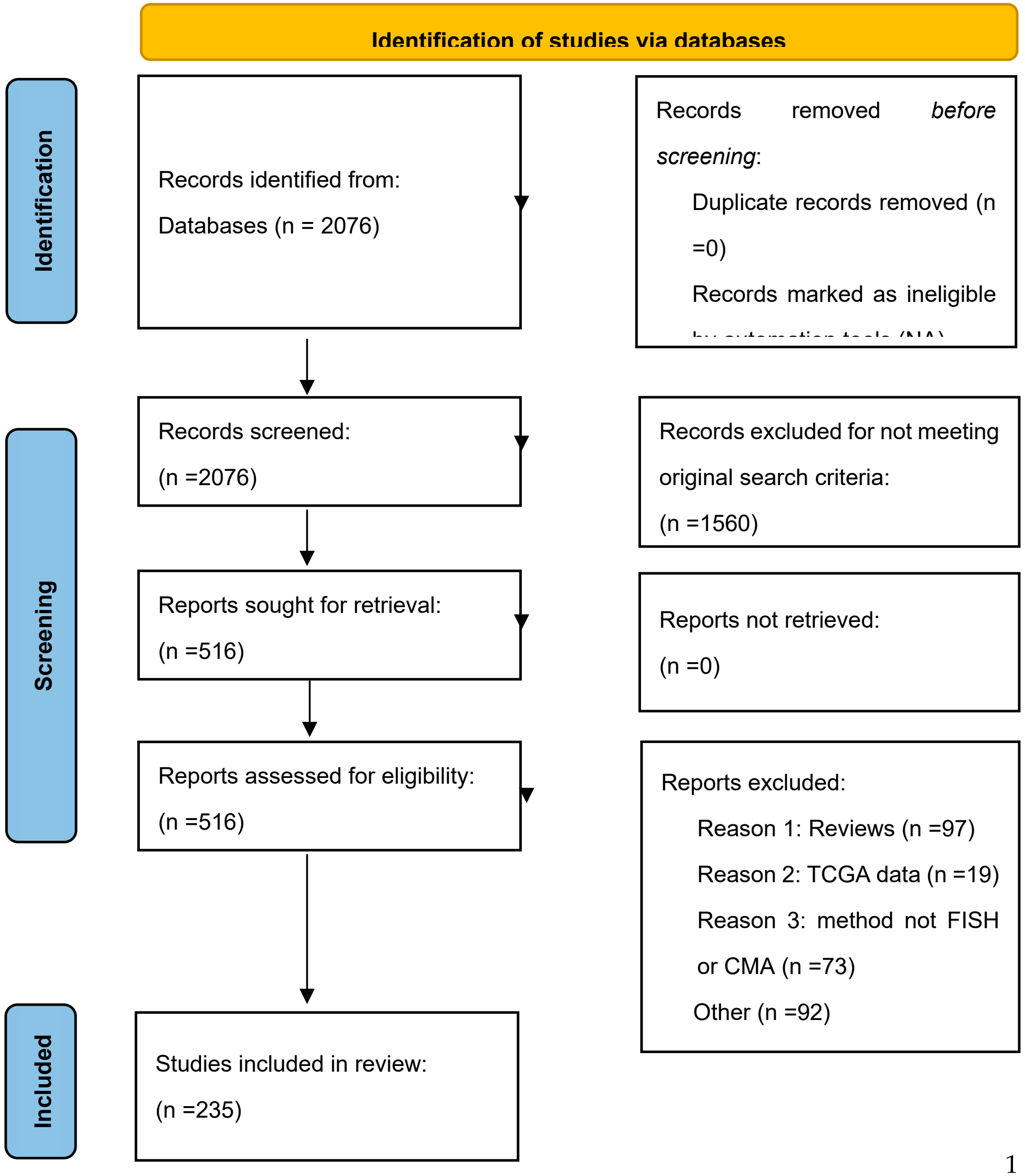

Supplementary Table S1: Rates of gains and losses across melanoma subtypes combined. P-values are from a two-sample test of whether there is a difference between gains and losses.

Melanoma subtype	Rate of Gains (%)	Rate of Losses (%)	P-value	Number of Cases
**General cutaneous**	38	30	<0.001	8680
**Acral**	33	7	<0.001	3007
**Blue nevus like**	34	37	0.656	176
**Desmoplastic**	34	3	0.002	35
**Mucosal**	14	8	<0.001	429
**Nevoid**	9	11	0.276	2934
**Spitzoid/Spitz**	30	25	0.093	667
**Uveal**	15	20	<0.001	28110

Supplementary Table S2: Rates of gains and losses for each chromosome in melanoma across all subtypes combined. P-values designate whether the rate of CNA is significantly higher than 5%. Significant values are in bold. Also shown are the number of manuscripts reporting results in CNAs for a given chromosome on which the rates are based. Also see Figure 2.

Chromosome	Rate of Gains (%)	P-value for Gains	Rate of Losses (%)	P-value for Losses	Number of manuscripts
1	**12**	<0.001	**19**	<0.001	57
2	**8**	0.009	3	0.847	10
3	1	1	**36**	<0.001	94
4	**17**	<0.001	5	0.5	25
5	**31**	<0.001	3	0.994	22
6	**21**	<0.001	**12**	<0.001	99
7	**31**	<0.001	1	1	42
8	**35**	<0.001	3	1	87
9	3	1	**57**	<0.001	71
10	1	1	**65**	<0.001	31
11	**25**	<0.001	**7**	<0.001	90

12	**19**	<0.001	1	1	21
13	**10**	<0.001	**7**	0.029	6
14	**29**	<0.001	**15**	<0.001	9
15	**16**	<0.001	**8**	0.035	11
16	2	1	**24**	<0.001	17
17	**16**	<0.001	**6**	<0.001	25
18	2	0.969	**11**	0.041	9
19	**26**	<0.001	2	0.729	7
20	**21**	<0.001	3	0.665	17
21	**11**	0.002	1	0.706	7
22	**20**	<0.001	2	0.998	5

References: [4 9-11 13-21 24-27 30 31 55 122-125 129-135 140 146-150 154-158 160 169-173 175-183 185-193 195 199 200 202 206-336]

Supplementary Table S3: Rates of chromosomal abnormalities detected in primary uveal melanoma associated with high risk of metastasis and aggressive clinical behavior.

Chromosome	Region	Abnormality	Rate of Abnormality (%)
1	1p	loss	27
1	1p36	loss	34
1	1q	gain	11
3	whole	loss	49
3	partial	loss	8
6	6q	loss	22
8	whole	gain	39
8	8p	loss	16
8	8p	gain	13
8	8q	gain	52
8	8q	isochromosome	23
8	8q24	gain	58
16	16q	loss	25
3, 8	3 whole, 8q	monosomy 3, gain 8q	43

References: [39-113 115-119 208] [39 41-49 51-55 60 61 64 68-70 72 74 76 77 82 84 86-89 92 93 96 99-101 104-109 111 115-119 208 337]

Supplementary Table S4: Rates of abnormalities for uveal primary and metastatic melanomas. P-values are for whether rates of the type of abnormality are significantly different between primary and metastatic uveal melanomas. Also shown are the number of manuscripts on which the rates are based. Values in red represent rates calculated from <25 reported cases.

Abnormality	Metastatic Melanoma (%)	Primary Melanoma (%)	P-value	Number of manuscripts
Gains	55	33	<0.001	109
Losses	56	16	<0.001	81
Polysomy	25	30	>0.999	45
Monosomy	62	48	0.008	156

Supplementary Table S5: Comparison of rates of abnormalities reported in Spitzoid lesions in three or more manuscripts. See also Figure 3. The symbol " -" designates data not available.

Region	Abnormality	Gene affected	Rate in Spitz nevus (%)	Rate in Spitz Melanocytoma (%)	Rate in Spitzoid/ Spitz Melanoma (%)	Number of manuscripts
6p25	gain	*RREB1*	3	9	54	13
6q23	loss	*MYB*	0	11	33	10
7q	gain	*BRAF*	2	67	21	3
8q	gain	*MYC*	-	3	4	3
9p21	loss	*CDKN2A*	2	18	39	21
11p15	gain	*HRAS*	19	0	4	10
11q13	gain	*CCND1*	0	6	33	11
FISH	at least one CNA	several	14	18	70	25
multiple by CMA	>3 CNAs	many	2	16	67	6

Supplementary Table S6: Rates of abnormalities for cutaneous primary and metastatic melanomas. P-values are for whether rates of the type of abnormality are significantly different between primary and metastatic melanomas. Also shown are the number of manuscripts on which the rates are based.

Abnormality	Metastatic Melanoma (%)	Primary Melanoma (%)	P-value	Number of manuscripts
FISH	84	82	0.909	69
>3 CNAs by CMA	99	94	0.125	48

Supplementary Table S7: CNAs reported in at least 3 manuscripts in at least 50 cases of primary melanomas and 50 cases of metastases with significant difference in rates (P-values given for differences). Individual P-values indicate whether each abnormality is significantly greater than 5%. Rates in bold were the greater of the comparison between primary and metastatic melanomas. See also figure 5.

Region	Abnormality	Genes affected	Rate in primary (%)	P-value primary	Rate in metastasis (%)	P-value metastasis	P-value difference	Number of primary melanoma cases	Number of metastasis cases
**3p13**	gain	*MITF*	10	<0.001	**19**	<0.001	0.009	255	214
**5p15**	gain	*TERT, NKD2*	**28**	<0.001	12	0.002	0.005	162	97
**6q**	loss		27	<0.001	**50**	<0.001	0.001	271	68
**chr7**	polysomy		25	<0.001	**57**	<0.001	<0.001	651	137
**7p11**	gain	*EGFR*	17	<0.001	**34**	<0.001	<0.001	231	213
**7q31**	gain	*MET, CAV1, others*	**32**	<0.001	17	<0.001	<0.001	348	195
**7q34**	gain	*BRAF*	30	<0.001	**58**	<0.001	<0.001	381	142
**8q24**	gain	*MYC*	**33**	<0.001	21	<0.001	0.008	567	160
**11q**	loss		24	<0.001	**40**	<0.001	0.014	225	68
**11q13**	gain	*CCND1*	**25**	<0.001	17	<0.001	<0.001	1629	379
**12q14**	gain	*CDK4*	**31**	<0.001	7	0.408	<0.001	322	129
**19p13**	Gain	*MAP2K2*	**44**	<0.001	4	1	<0.001	137	69

Supplementary Table S8: CNAs reported in at least 3 manuscripts in at least 50 cases of primary melanomas and 50 cases of metastases with NO significant difference in rates (P-values given for differences). Individual P-values indicate whether each abnormality is significantly greater than 5%. See also Figure 6.

**Region**	**Abnormality**	**Genes**	**Rate in primary (%)**	**P-value primary**	**Rate in metastasis (%)**	**P-value metastasis**	**P-value difference**	**Number of primary melanoma cases**	**Number of metastasis cases**
**1p12**	gain	*NOTCH2, ADAM30*	13	0	22	0	0.066	137	129
**1p13**	gain	*NRAS*	15	0	10	0.002	0.275	162	175
**1p36**	loss	*PRDM16, ARID1A + others*	34	0	35	0	0.939	171	114
**4q12**	gain	*KIT, KDR, PDGFRA*	6	0.659	5	1	0.99	411	156
**7p**	gain		22	0	18	0	0.322	362	140
**9p21**	loss	*CDKN2A, CDKN2B*	52	0	51	0	0.884	880	168
**chr10**	monosomy		44	0	46	0	0.845	325	76
**10q23**	loss	*PTEN*	32	0	36	0	0.48	218	129
**11q14**	gain	*GAB2*	9	0.013	16	0	0.148	212	68
**12p12**	gain	*KRAS, PIK3C2G*	9	0.021	7	0.562	0.809	162	69
**12q15**	gain	*MDM2, others*	9	0.001	5	1	0.1	364	251
**20q13**	gain	*GNAS, others*	27	0	26	0	1	226	80

Supplementary Table S9: Abnormalities reported in various benign nevus subtypes. The 95% confidence intervals for each proportion of the abnormality are given. The symbol " -" designates data not available.

Nevus subtype	Abnormality	95% CI for the Proportion of cases	Significantly >5%
General cutaneous nevi	Gains	1.9-3.1	no
	Losses	0.1-0.6	no
	FISH	4.1-6.2	no
	>3 CNAs by CMA	0.3-6.2	no
Blue nevi	Gains	1.9-3.1	no
	Losses	0.1-0.6	no
	FISH	4.1-6.2	no
	>3 CNAs by CMA	-	no
Spitz nevi	Gains	1.9-3.1	no
	Losses	0.1-0.6	no
	FISH	4.1-6.2	no
	>3 CNAs by CMA	0.3-6.2	no

Supplementary Table S10: FISH probe sets for analysis of melanocytic lesions with published data included in this study.

Number of probes	Chromosomes	Loci	Genes
**4**	6, 11	6p25, 6q23, CEP6, 11q13	*RREB1, MYB, CCND1*
**5**	6, 8, 9, 11	6p25, 8q24, 9p21, CEP9, 11q13	*RREB1, MYC, CDKN2A, CCND1*
**6**	6, 9, 11	6p25, 6q23, CEP6, 9p21, CEP9, 11q13	*RREB1, MYB, CDKN2A, CCND1*
**6**	6, 8, 9, 11	6p25, 6q23, CEP6, 8q24, 9p21, 11q13	*RREB1, MYB, MYC, CDKN2A, CCND1*

**8**	6, 8, 9, 11	6p25, 6q23, 8q24, 8p11.1, 9p21, 9q21.2, 11q13, 11p15.5	*RREB1, MYB, MYC, POETA, CDKN2A, GNAQ, CCND1, HRAS*

Supplementary Table S11: Genes classified as other/complex in Table 1.

Gene	Function/Potential Role
ADAM30	Limited functional evidence supporting a driver role in melanoma
BPTF	Chromatin remodeler with context-dependent oncogenic properties
CYP24	Vitamin D metabolism gene; indirect relevance to tumor biology
EP300	Histone acetyltransferase; may function as coactivator or tumor suppressor depending on context
KIRREL	Limited mechanistic validation as melanoma driver
MKL1	Transcriptional coactivator; context-dependent oncogenic activity
NOTCH2	Context-dependent signaling with oncogenic and tumor-suppressive roles depending on cellular context
PDE11A	Phosphodiesterase with unclear contribution to melanoma progression
PDE4DIP	Scaffold protein; no consistent evidence of recurrent oncogenic activation in melanoma
PHIP	Implicated in melanoma progression but mechanistically complex and not a canonical oncogene
PIK3C2G	PIK3 family member; limited evidence of recurrent activating alterations in melanoma
S100A9, S100A10, S100A11, S100A12	Inflammatory mediators more commonly implicated in tumor microenvironment modulation than as primary genomic drivers
SS18L1	Transcriptional regulator without clear melanoma driver validation

Supplementary Table S12: Genes classified as other/complex in Table 2.

Gene	Function/Potential Role
CALML5	Calcium-binding protein; limited oncogenic validation
CD274 (PD-L1)	Immune checkpoint regulator; deletion effects are context-dependent
CDK10	Cell-cycle regulator; limited melanoma-specific driver evidence
CHEK1	DNA damage response kinase; dual context-dependent role
ETS1	Transcription factor with context-dependent oncogenic properties
IL15RA	Immune regulatory receptor; indirect tumor role
JAK2	Oncogenic kinase; loss not typical driver event in melanoma
LARP4B	RNA-binding protein; insufficient evidence as melanoma driver
MYB	Canonical oncogene; loss does not represent typical driver mechanism in melanoma
NET1	RhoA GEF; limited melanoma-specific evidence
PRDM16	Context-dependent transcriptional regulator; not established as recurrent melanoma tumor suppressor
PRKCQ	Kinase with signaling roles; melanoma-specific driver role unclear
YAP1	Hippo pathway oncogene; deletion suggests complex regional effects

Supplementary Table S13: Literature search strategy detail through Ovid. Database(s): EBM Reviews - Cochrane Central Register of Controlled Trials July 2022, EBM Reviews - Cochrane Database of Systematic Reviews 2005 to 31 August 2022, Embase 1974 to 2 September 2022, Ovid MEDLINE(R) and Epub Ahead of Print, In-Process, In-Data-Review & Other Non-Indexed Citations, Daily and Versions 1946 to 2 September 2022.

	Searches	Results
**1**	exp Melanoma/	291329
**2**	exp Melanocytes/	34516
**3**	exp Nevus/	40717
**4**	(melanocyte* or melanocytic or melanocytoma* or melanoma* or MELTUMP or naevi or naevus or nevi or nevus or spitz* or STUMP).ti,ab,kw.	419143
**5**	1 or 2 or 3 or 4	488612
**6**	exp DNA Copy Number Variations/	43243
**7**	exp Comparative Genomic Hybridization/	26503

**8**	exp In Situ Hybridization, Fluorescence/	128041
**9**	(CGH or "chromosomal aberration*" or "chromosomal alteration*" or "chromosomal amplification*" or "chromosomal deletion*" or "chromosomal duplication*" or CNA or CNV or "comparative genomic hybridization*" or "copy number" or "FISH assay*" or "fluorescence in situ hybridisation*" or "fluorescence in situ hybridization*" or "fluorescent in situ hybridisation*" or "fluorescent in situ hybridization*" or "Single Nucleotide Polymorphism array*" or "SNP array*").ti,ab,kw.	275164
**10**	or/6-9	345114
**11**	5 and 10	5670
**12**	(case* adj3 report*).mp,pt.	5513377
**13**	11 not 12	4747
**14**	(exp animals/ or exp nonhuman/) not exp humans/	11921774

**15**	(alpaca or alpacas or amphibian or amphibians or animal or animals or antelope or armadillo or armadillos or avian or baboon or baboons or beagle or beagles or bee or bees or bird or birds or bison or bovine or buffalo or buffaloes or buffalos or "c elegans" or "Caenorhabditis elegans" or camel or camels or canine or canines or carp or cats or cattle or chick or chicken or chickens or chicks or chimp or chimpanze or chimpanzees or chimps or cow or cows or "D melanogaster" or "dairy calf" or "dairy calves" or deer or dog or dogs or donkey or donkeys or drosophila or "Drosophila melanogaster" or duck or duckling or ducklings or ducks or equid or equids or equine or equines or feline or felines or ferret or ferrets or finch or finches or fish or flatworm or flatworms or fox or foxes or frog or frogs or "fruit flies" or "fruit fly" or "G mellonella" or "Galleria mellonella" or geese or gerbil or gerbils or goat or goats or goose or gorilla or gorillas or hamster or hamsters or hare or hares or heifer or heifers or horse or horses or insect or insects or jellyfish or kangaroo or kangaroos or kitten or kittens or lagomorph or lagomorphs or lamb or lambs or llama or llamas or macaque or macaques or macaw or macaws or marmoset or marmosets or mice or minipig or minipigs or mink or minks or monkey or monkeys or mouse or mule or mules or nematode or nematodes or octopus or octopuses or orangutan or "orang-utan" or orangutans or "orang-utans" or oxen or parrot or parrots or pig or pigeon or pigeons or piglet or piglets or pigs or porcine or primate or primates or quail or rabbit or rabbits or rat or rats or reptile or reptiles or rodent or rodents or ruminant or ruminants or salmon or sheep or shrimp or slug or slugs or swine or tamarin or tamarins or toad or toads or trout or urchin or urchins or vole or voles or waxworm or waxworms or worm or worms or xenopus or "zebra fish" or zebrafish)	10174409

	not (human or humans or patient or patients)).ti,ab,hw,kw.	
**16**	13 not (14 or 15)	4454
**17**	limit 16 to english language [Limit not valid in CDSR; records were retained]	4370
**18**	limit 17 to yr="1998 -Current"	4170

**19**	limit 18 to (letter or conference abstract or editorial or erratum or note or addresses or autobiography or bibliography or biography or blogs or comment or dictionary or directory or interactive tutorial or interview or lectures or legal cases or legislation or news or newspaper article or overall or patient education handout or periodical index or portraits or published erratum or video-audio media or webcasts) [Limit not valid in CCTR,CDSR,Embase,Ovid MEDLINE(R),Ovid MEDLINE(R) Daily Update,Ovid MEDLINE(R) PubMed not MEDLINE,Ovid MEDLINE(R) In-Process,Ovid MEDLINE(R) Publisher; records were retained]	980
**20**	18 not 19	3190
**21**	remove duplicates from 20	2076

PRISMA

PRISMA 2020 Checklist

Supplementary Table S14: PRISMA 2020 Checklist.

Section and Topic	Item #	Checklist item	Location where item is reported
**TITLE**			
Title	1	Identify the report as a systematic review.	Title page
**ABSTRACT**			
Abstract	2	See the PRISMA 2020 for Abstracts checklist.	Title page/abstract
**INTRODUCTION**			
Rationale	3	Describe the rationale for the review in the context of existing knowledge.	1. Introduction
Objectives	4	Provide an explicit statement of the objective(s) or question(s) the review addresses.	Abstract
**METHODS**			
Eligibility criteria	5	Specify the inclusion and exclusion criteria for the review and how studies were grouped for the syntheses.	2. Methods
Information sources	6	Specify all databases, registers, websites, organisations, reference lists and other sources searched or consulted to identify studies. Specify the date when each source was last searched or consulted.	2. Methods, Supplementary Figure 1, Supplementary Table S13
Search strategy	7	Present the full search strategies for all databases, registers and websites, including any filters and limits used.	2. Methods, Supplementary Figure 1, Supplementary Table S13
Selection process	8	Specify the methods used to decide whether a study met the inclusion criteria of the review, including how many reviewers screened each record and each report retrieved, whether they worked independently, and if applicable, details of automation tools used in the process.	2. Methods, Supplementary Figure 1, Supplementary Table S13
Data collection process	9	Specify the methods used to collect data from reports, including how many reviewers collected data from each report, whether they worked independently, any processes for obtaining or confirming data from study investigators, and if applicable, details of automation tools used in the process.	2. Methods, Supplementary Figure 1, Supplementary Table S13
Data items	10a	List and define all outcomes for which data were sought. Specify whether all results that were compatible with each outcome domain in each study were sought (e.g. for all measures, time points, analyses), and if not, the methods used to decide which results to collect.	2. Methods
	10b	List and define all other variables for which data were sought (e.g. participant and intervention characteristics, funding sources). Describe	2. Methods

Section and Topic	Item #	Checklist item	Location where item is reported
		any assumptions made about any missing or unclear information.	
Study risk of bias assessment	11	Specify the methods used to assess risk of bias in the included studies, including details of the tool(s) used, how many reviewers assessed each study and whether they worked independently, and if applicable, details of automation tools used in the process.	2. Methods
Effect measures	12	Specify for each outcome the effect measure(s) (e.g. risk ratio, mean difference) used in the synthesis or presentation of results.	2. Methods
Synthesis methods	13a	Describe the processes used to decide which studies were eligible for each synthesis (e.g. tabulating the study intervention characteristics and comparing against the planned groups for each synthesis (item #5)).	2. Methods
	13b	Describe any methods required to prepare the data for presentation or synthesis, such as handling of missing summary statistics, or data conversions.	2. Methods
	13c	Describe any methods used to tabulate or visually display results of individual studies and syntheses.	2. Methods
	13d	Describe any methods used to synthesize results and provide a rationale for the choice(s). If meta-analysis was performed, describe the model(s), method(s) to identify the presence and extent of statistical heterogeneity, and software package(s) used.	2. Methods
	13e	Describe any methods used to explore possible causes of heterogeneity among study results (e.g. subgroup analysis, meta-regression).	2. Methods
	13f	Describe any sensitivity analyses conducted to assess robustness of the synthesized results.	2. Methods
Reporting bias assessment	14	Describe any methods used to assess risk of bias due to missing results in a synthesis (arising from reporting biases).	2. Methods
Certainty assessment	15	Describe any methods used to assess certainty (or confidence) in the body of evidence for an outcome.	2. Methods
**RESULTS**			
Study selection	16a	Describe the results of the search and selection process, from the number of records identified in the search to the number of studies included in the review, ideally using a flow diagram.	2. Methods, Supplementary Figure 1, Supplementary Table S13
	16b	Cite studies that might appear to meet the inclusion criteria, but which were excluded, and explain why they were excluded.	Supplementary Figure 1
Study characteristics	17	Cite each included study and present its characteristics.	2. Methods, Supplementary Figure 1, Supplementary Table S13
Risk of bias in studies	18	Present assessments of risk of bias for each included study.	2. Methods, Supplementary

Section and Topic	Item #	Checklist item	Location where item is reported
			Figure 1, Supplementary Table S13
Results of individual studies	19	For all outcomes, present, for each study: (a) summary statistics for each group (where appropriate) and (b) an effect estimate and its precision (e.g. confidence/credible interval), ideally using structured tables or plots.	N/A
Results of syntheses	20a	For each synthesis, briefly summarise the characteristics and risk of bias among contributing studies.	N/A
	20b	Present results of all statistical syntheses conducted. If meta-analysis was done, present for each the summary estimate and its precision (e.g. confidence/credible interval) and measures of statistical heterogeneity. If comparing groups, describe the direction of the effect.	N/A
	20c	Present results of all investigations of possible causes of heterogeneity among study results.	N/A
	20d	Present results of all sensitivity analyses conducted to assess the robustness of the synthesized results.	N/A
Reporting biases	21	Present assessments of risk of bias due to missing results (arising from reporting biases) for each synthesis assessed.	N/A
Certainty of evidence	22	Present assessments of certainty (or confidence) in the body of evidence for each outcome assessed.	N/A
**DISCUSSION**			
Discussion	23a	Provide a general interpretation of the results in the context of other evidence.	3.11 Other Ancillary Techniques, 4. Discussion
	23b	Discuss any limitations of the evidence included in the review.	4. Discussion
	23c	Discuss any limitations of the review processes used.	4. Discussion
	23d	Discuss implications of the results for practice, policy, and future research.	4. Discussion, 5. Evidence-based Recommendations, Figure 7
**OTHER INFORMATION**			
Registration and protocol	24a	Provide registration information for the review, including register name and registration number, or state that the review was not registered.	Statement provided on page 21 (the review is not registered).
	24b	Indicate where the review protocol can be accessed, or state that a protocol was not prepared.	Statement provided on page 21 (Data Availability)

PRISMA 2020 Checklist

Section and Topic	Item #	Checklist item	Location where item is reported
	24c	Describe and explain any amendments to information provided at registration or in the protocol.	N/A
Support	25	Describe sources of financial or non-financial support for the review, and the role of the funders or sponsors in the review.	Statement provided on page 21 (Funding)
Competing interests	26	Declare any competing interests of review authors.	Statement provided on page 21 (COI)
Availability of data, code and other materials	27	Report which of the following are publicly available and where they can be found: template data collection forms; data extracted from included studies; data used for all analyses; analytic code; any other materials used in the review.	Statement provided on page 21 (Data Availability)

From: Page MJ, McKenzie JE, Bossuyt PM, Boutron I, Hoffmann TC, Mulrow CD, et al. The PRISMA 2020 statement: an updated guideline for reporting systematic reviews. BMJ 2021;372:n71. doi: 10.1136/bmj.n71. This work is licensed under CC BY 4.0. To view a copy of this license, visit https://creativecommons.org/licenses/by/4.0/
